# Identity trust management based on differential privacy for internet of vehicles

**DOI:** 10.1038/s41598-026-50273-z

**Published:** 2026-05-06

**Authors:** Liang Ren, Hui Li, Yutong Liu, Dan Liao, Ming Zhang, Haiyan Jin

**Affiliations:** 1 University of Electronic Science and Technology of China, Chengdu, China; 2 Jiangxi Communication Terminal Industry Technology Research Institute Co., LTD, Ji’an, China; 3 Jiangxi Xingtai Technology Co., LTD, Ji’an, China

**Keywords:** Trust management, Intra-domain vehicle, Identity security, Differential privacy, Engineering, Mathematics and computing

## Abstract

Vehicle trust management is closely related to the identity security of intra-domain vehicles and has garnered widespread attention. However, there is a significant conflict between vehicle trust and identity privacy, which has led to the emergence of novel trust link attack issues. To address the problem, this paper proposes a vehicle identity trust management algorithm based on differential privacy, called DITDP. In DITDP, we first design an identity trust evaluation model based on D-S evidence, which is used to quantify vehicle trust values, including the direct trust, the recommended trust, and the aggregated trust. Then, we design the dynamic trust evaluation and identity privacy protection modules in DITDP. The dynamic trust evaluation module corrects conflict between vehicle trust components and can dynamically update vehicle trust values in the time domain. The identity privacy protection module achieves indistinguishable trust while ensuring the availability of trust value by using Differential privacy. Finally, the simulations verify that the DITDP algorithm performs well in terms of identity protection ability, trust availability, and other aspects.

## Introduction

To facilitate the management and guidance of vehicles, the Internet of Vehicles (IoV) can be divided into multiple vehicle domains. The vehicle domain is a collection of vehicles registered with the same trusted entity (such as a certificate authority) and managed by that entity. The intra-domain vehicle communication can be managed with the involvement of a trusted entity. There is a certain degree of communication interaction history in vehicle domain^1^. Thus, the identity security is fundamental for the intra-domain vehicles.

Nowadays, to ensure identity security and recognize the malicious vehicles, various types of vehicle identity trust management schemes have been proposed for intra-domain vehicles^2^. In existing trust management schemes, identity privacy issues are rarely considered as an additional factor; instead, the focus is primarily on metrics such as the accuracy of trust evaluation. In other words, the protection of vehicle identity privacy is merely implied in the background and is not thoroughly integrated or elaborated upon in the trust management schemes. However, in practical scenarios, solutions for privacy and trust can mutually influence each other. To achieve identity privacy protection, many solutions revolve around pseudonym mechanisms, among which the most common is to use pseudonyms as virtual unique identifiers for nodes, and these identifiers are changed periodically. Whether it is a distributed or a centralized pseudonym replacement mechanism, the pseudonym of vehicle changes at a certain moment or when it reaches a certain eligible location. In this context, the vehicles’ locations are macroscopically the same, making it difficult for attackers to differentiate them based on positional differences or driving information such as speed and direction. However, with the introduction of trust management schemes, trust values become a new public identifiable factor for vehicles. The linkability of trust undermines the unlinkability of pseudonyms, thus compromising the effectiveness of identity privacy protection. In simple terms, even if a vehicle’s pseudonym changes, the trust value may remain the same.

**Fig. 1:**
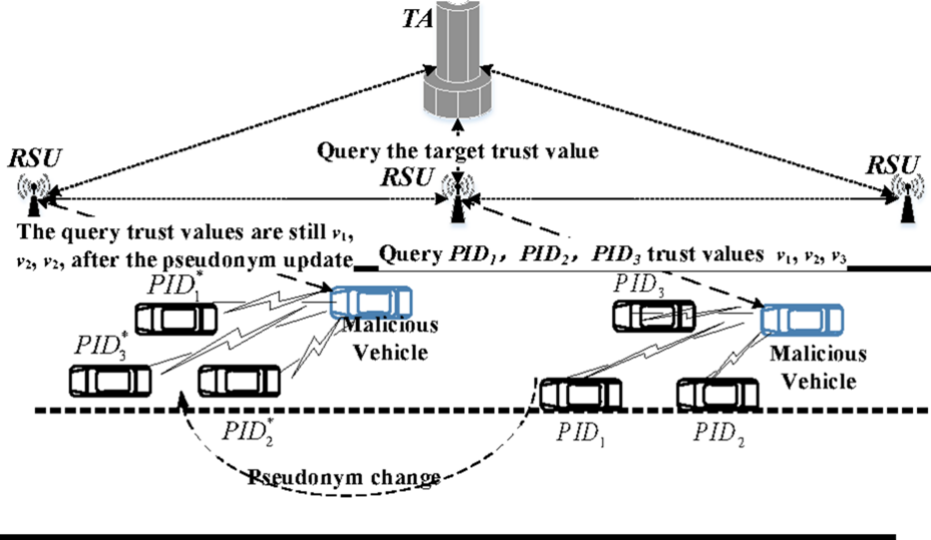
Trust link attack model.

The trust value of the target vehicle is an important reference for other vehicles^3^. It will be used to judge the trustworthiness of the target vehicle. But the existence of trust value will make the pseudonym system have the risk of failure, which brings the identity leakage hidden danger. The trust link attack model is as shown in Fig. 1. Suppose that the vehicles $\:PI{D}_{1}$, $\:PI{D}_{2}$, and $\:PI{D}_{3}$ are close to each other at a certain time. PID is the unique pseudonym identifier of the vehicle. And the attacker gets their trust values from the Road Side Unit (RSU) as $\:{v}_{1}$, $\:{v}_{2}$, and $\:{v}_{3}$. If these three vehicles undergo a pseudonym change for a short period of time in a particular case, their respective new pseudonyms are $\:PI{D}_{1}^{*}$, $\:PI{D}_{2}^{*}$, and $\:PI{D}_{3}^{*}$. Then the attacker queries their trust values from the RSU again as $\:{v}_{1}$, $\:{v}_{2}$, and $\:{v}_{3}$. At this point, the pseudonym change fails and the attacker can still identify and track them. In these cases, the attacker can easily identify the vehicle by comparing the trust values before and after the pseudonym change. Therefore, the existing trust management mechanisms can lead to conflicting issues between vehicle trust values and identity privacy protection.

Therefore, to address the trust link attack problem, this paper proposes a vehicle identity trust management algorithm based on differential privacy for intra-domain vehicles, called DITDP. DITDP performs the calculation of vehicle trust values within the vehicular domain. At the same time, by utilizing differential privacy, it releases the perturbed trust values to the public in an indistinguishable manner, while the true trust values are kept hidden. This approach protects the privacy of vehicle identities, preventing attackers from deciphering a vehicle’s pseudonym through the perturbed trust values. The main contributions of this paper are summarized as follows:

1) An identity trust evaluation model is proposed based on D-S evidence. The vehicle quantifies the direct trust value of the target vehicle through its historical behavior. If the direct trust value cannot adequately judge the trustworthiness of the target vehicle, the direct trust values of other vehicles to the target vehicle is used as trust components to calculate the recommended trust value. Then, the aggregated trust value of target vehicle is obtained by aggregating recommended trust value based on the *Dempster-Shafer* synthesis law.

2) Based on the identity trust evaluation model, the *DITDP* algorithm is designed, which consists of two main modules: dynamic trust assessment and identity privacy protection. Dynamic trust assessment module enables the correction of conflicts between individual trust components. It reduces the impact of malicious trust components on the final aggregated trust value of a vehicle. Identity privacy protection module achieves the discriminability of vehicle trust by differential privacy.

The rest of this paper will be arranged as follows: In Sect.  2, we have summarized the centralized and distributed trust management schemes; In Sect.  3, we built the identity trust evaluation model to calculate the direct trust, recommended trust and aggregated trust; In Sect.  4, the *DITDP* algorithm is designed in detail. In Sect.  5, the performance of *DITDP* algorithm and other algorithms have been compared through simulation; Finally, the conclusion of this paper is presented in Sect.  6.

## Related work

In recent years, to cope with various identity security issues in IoV, some trust management schemes have been proposed by domestic and international scholars, which can be mainly classified into centralized and distributed schemes^4,5^.

### Centralized trust management

Han et al.^6^ proposed a dynamic trust-management framework that combines graph-based sharding with blockchain techniques to improve scalability of reputation aggregation in IoV; Ren^7^ proposed a dynamic trust-based scheme that evaluates node trust values to enhance security and reliability in in-vehicle networks; Sehar et al.^8^ demonstrated that consortium/permissioned blockchain frameworks could significantly improve misbehavior detection rates in vehicular reputation systems while highlighting scalability trade-offs; Cui et al.^9^ developed a reputation-based message authentication framework for 5G-enabled vehicles.

### Distributed trust management

In a scenario with a high degree of distribution like vehicular networking, it is not possible to monitor all nodes internally and announce their behavioral characteristics through a central authority. This is why distributed trust management have been widely studied. For example, Yang et al.^10^ proposed a distributed vehicle trust assessment algorithm. It analyzed the trust relations of vehicles through vehicle social networks; Wang et al.^11^ proposed a trust management scheme where vehicles must obtain a collection of pseudonyms from the system. Since trust values are limited, vehicles would still be tracked if there is a limit on the number of vehicles in the RSU area; Inedjaren et al.^12^ proposed a decentralized blockchain-based trust management system. It assessed the trustworthiness of nodes based on their routing performance; Wang et al.^13^ proposed a trust management system based a deep learning verification model; Liu et al.^14^ proposed a conditional privacy protection authentication scheme using layered kana in IoV, based on the Elliptic Curve Diffie Hellman problem; Chanchal et al.^15^ proposed a secure, efficient, and conditional privacy anonymous batch authentication scheme based on elliptic curve cryptography; Cheng et al.^16^ designed an identity based signature scheme and a blockchain assisted pseudonym management scheme; Mahmoud et al.^17^ proposed a blockchain based reputation system; Parametsworath et al.^18^ proposed a V2G network authentication key exchange protocol based on decentralized identifiers; Hou et al.^19^ proposed a scalable reservation authentication and key agreement protocol for 5G-V2G systems; Miao et al.^20^ proposed a cross-domain authentication scheme for V2G networks in smart grid systems based on consortium blockchain; Li et al.^21^ proposed an identity management framework based on CP-ABE, which protected the identity privacy of edge entities by encrypting classified identity information.

### Comparative analysis

Table 1 summarizes the key differences in performance, privacy protection, and computational overhead between existing trust management schemes and the proposed DITDP.

1. Centralized trust schemes rely on a central authority to manage trust and often lack robustness against single points of failure and scalability issues. While some schemes may briefly consider providing privacy through identity-based encryption or pseudonymization, they remain vulnerable to trust link attacks if trust values are not adequately protected. While DITDP uses a distributed architecture in which every vehicle can calculate its trust value without a central authority.

(2) Distributed trust schemes can improve scalability and robustness but often struggle to ensure consistent trust evaluations across the network and have poor privacy guarantees. DITDP enhances the privacy of the data by perturbing the trust value via the “Staircase Algorithm”, based on differential privacy. Specifically, DITDP incorporates a dynamic trust evaluation process that adapts to changing vehicle behavior and network conditions. This dynamic adaptation ensures that the trust values accurately reflect the current trustworthiness of the vehicles while providing identity privacy.

**Table 1:** The comparison of different approaches.

Feature	Centralized Trust Management	Distributed Trust Management	DITDP
Performance	Limited by central server	Scalable	Scalable, leverages distributed computation
Privacy Protection	Vulnerable to central attacks	Limited	Strong, using differential privacy
Trust Link Attack Resistance	Low	Low	High, mitigates trust link attacks through perturbation
Computational Overhead	High at central server	Distributed	Moderate, trades off computation for privacy
Robustness	Low, single point of failure	High	High, distributed with privacy preserving mechanisms
Scalability	Limited by central server	High	High, designed for large-scale vehicular networks

## Identity trust evaluation model

### Model establishment

**Fig. 2:**
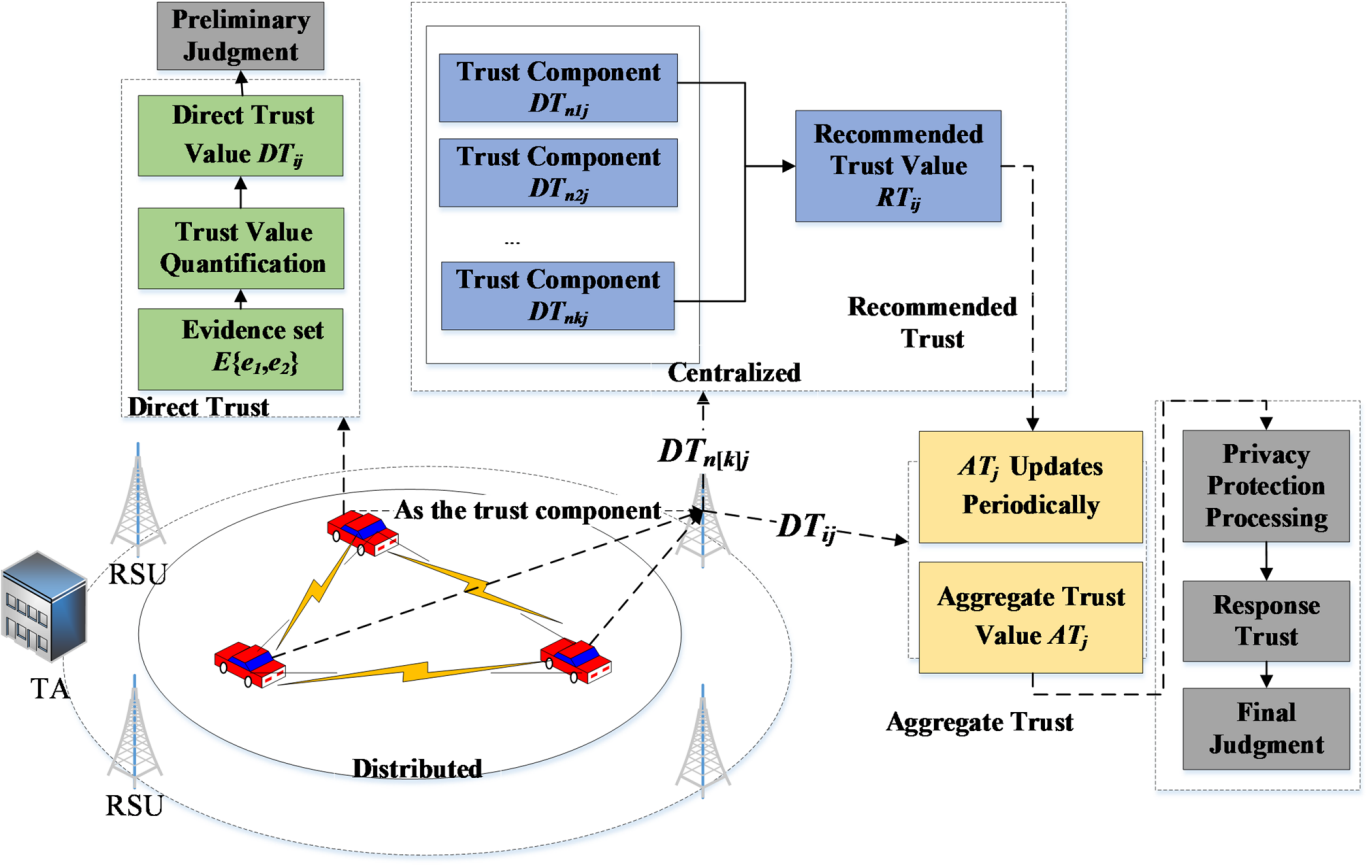
Identity trust evaluation model.

The identity trust evaluation model is shown in Fig. 2. The proposed model leverages aspects of both distributed and centralized approaches to trust management. It utilizes distributed trust evaluation among vehicles for direct trust assessment, while employing a centralized entity (the RSU) for aggregating and disseminating trust information. This hybrid approach combines the benefits of scalability from distributed systems with the reliability and consistency of centralized systems.

The identity trust evaluation model is mainly quantified based on the behavior and information of the target vehicle, including the direct trust, the recommended trust, and the aggregated trust. As shown in Fig. 2, the direct trust $\:D{T}_{ij}$ and recommended trust $\:R{T}_{ij}$ are combined to form the vehicle aggregated trust $\:A{T}_{j}$, which is the final result of the vehicle trust evaluation.

In Fig. 2, we define a multivariate vehicle theoretical model $\:<\varOmega\:,\:E,m\left(A\right),Pl,d\left({m}_{1},{m}_{2}\right)>$ to illustrate the principle of vehicle trust evaluation.

1) Vehicle identification framework $\:{\Omega\:}$: $\:{\Omega\:}$ is a finite set consisting of *N* mutually exclusive and exhaustive propositions. It can be expressed as $\:{\Omega\:}=\left\{{A}_{1},{A}_{2},\dots\:,{A}_{N}\right\}$, where $\:{A}_{i}$ represents each exclusive events. In this paper, three exhaustive events in the vehicle trust evaluation are set. The first event $\:{A}_{1}$ represents the target vehicle as credible. The second event $\:{A}_{2}$ represents the target vehicle as untrustworthy, that is, the malicious node. The third event $\:{A}_{3}\:$represents the inability to determine whether the target vehicle is credible based on the current evidence sample. Therefore, the vehicle identification framework can be expressed as $\:\varOmega\:=\{{A}_{1},{A}_{2},{A}_{3}\}$.

2) Vehicle evidence collection $\:E$: It is a basis to evaluate the behavior of the vehicle. We consider two kinds of evidence $\:E\left\{{e}_{1},\:{e}_{2}\right\}$ based on the historical interaction. The evidence $\:{e}_{1}$ represents the proportion of reliable or false messages sent by the evaluated vehicle. And evidence $\:{e}_{2}$ represents the time delay in the response packet of the evaluated vehicle.

3) Event probability function $\:m\left({A}_{i}\right)$: It is the basic confidence function on the power set $\:P\left(\varOmega\:\right)$, representing the set of all possible subsets of Ω, including the empty set and Ω itself. We use $\:{m}^{{e}_{i}}\left(A\right)$ to denote the occurrence probability of the three independent events on evidence $\:{e}_{i}$. And $\:BPA$ represents the set of basic probability functions.

1 $$\left\{ \begin{gathered} {m^{{e_i}}}{\mathrm{(}}{A_1}{\mathrm{)}}={{\mathrm{a}}_i},{m^{{e_i}}}{\mathrm{(}}{A_{\mathrm{2}}}{\mathrm{)}}={{\mathrm{b}}_i},{m^{{e_i}}}{\mathrm{(}}{A_{\mathrm{3}}}{\mathrm{)}}={{\mathrm{c}}_i},\quad i=\{ 1,2\} \hfill \\ \sum\limits_{{A \subseteq \Omega }} {{m^{{e_i}}}(A){\mathrm{=}}1,\quad i=\{ 1,2\} } \hfill \\ BPA=\{ {m^{{e_1}}}{\mathrm{(}}{A_1}{\mathrm{)}},{m^{{e_1}}}{\mathrm{(}}{A_2}{\mathrm{)}},{m^{{e_1}}}{\mathrm{(}}{A_3}{\mathrm{),}}{m^{{e_2}}}{\mathrm{(}}{A_1}{\mathrm{)}},{m^{{e_2}}}{\mathrm{(}}{A_2}{\mathrm{)}},{m^{{e_2}}}{\mathrm{(}}{A_3}{\mathrm{)}}\} \hfill \\ \end{gathered} \right.$$

4) Event non-denial function $\:Pl\left({A}_{i}\right)$: It means the degree of non-denial of the $\:{A}_{i}$. In this paper, we use $\:Pl\left({A}_{3}\right)$ to represent the magnitude of the probability of occurrence of the event $\:{A}_{3}$ for which the vehicle confidence level cannot be judged.

2 $$Pl({A_3})=1 - {m^{{e_1}}}({A_1}) - {m^{{e_2}}}({A_2})$$

5) Trusted Component Distance $\:d\left({m}_{i},{m}_{j}\right)$: In this paper, the ‘trust component’ refers to the direct trust value that a given vehicle assigns to another vehicle, based on its observed behavior^22^. This direct trust value serves as a building block for calculating the recommended trust and aggregated trust values. The trusted Component Distance $\:d$ represents the degree of conflict between the vehicle trust components $\:{m}_{i}={\left({m}_{i}\left({A}_{1}\right),{m}_{i}\left({A}_{2}\right),{m}_{i}\left({A}_{3}\right)\right)}^{T}$ and $\:{m}_{j}\:=\:{\left(\:{m}_{j}\left({A}_{1}\right),\:\:{m}_{j}\left({A}_{2}\right),\:\:{m}_{j}\left({A}_{3}\right)\:\right)}^{T}$ under $\:{\Omega\:}$. We use it to represent the conflict situation between any two different trust components $\:{m}_{i}$ and $\:{m}_{j}$ among $\:k$ vehicle trust components, that is, the consistency between the trust components. And we define the *Josselme* trust distance $\:d\left({m}_{1},{m}_{2}\right)$^12^ between $\:{m}_{i}$ and $\:{m}_{j}$ as Eq. (3).

3 $$d({m_i},{m_j})=\sqrt {\frac{1}{2}{{({m_i} - {m_j})}^T}D({\mathfrak{m}_i} - {m_j})}$$

Where $\:D$ is a 3 × 3 matrix with rows corresponding to the evidence vector *m*_*i*_ and columns corresponding to the trust component $\:{m}_{j}$. This matrix is specifically designed for the three possible outcomes in our trust evaluation model: credible (*A*_1_), untrustworthy (*A*_2_), and inability to determine (*A*_3_). The *d*(*m*_1_, *m*_2_) is computed by considering the difference in belief assigned to each possible outcome (*A*_1_, *A*_2_, and *A*_3_ in our case) by the trust components being compared. Specifically, for each possible outcome, we calculate the absolute difference in the belief values assigned by the two components. These absolute differences are then combined using a root-mean-square approach, resulting in a single value representing the overall distance between the two trust components. The Trusted Component Distance formula is designed to capture the degree of conflict between two trust components by considering the differences in their probability assignments for each possible event. The absolute value of the differences ensures that both positive and negative deviations contribute to the overall distance. The square root is used to normalize the distance and provide a more intuitive scale. The elements in $\:D$ can be expressed as Eq. (4), where $\:\left|T\right|$ is the base of the set $\:T$, indicating the number of elements in the set $\:T$. $\:{A}_{i}$ and $\:{B}_{j}$ are the elements in $\:P\left(\varOmega\:\right)$.

4 $${d_{ij}}=\left| {\frac{{{A_i} \cap {B_j}}}{{{A_i} \cup {B_j}}}} \right|$$

However, in this model, the basic belief assignment is allocated only to the singleton subsets of Ω (i.e., {*A*_1_}, {*A*_2_}, and {*A*_3_}). Under this specific condition, the general *Jousselme* distance between two trust components simplifies to a scaled Euclidean distance. Therefore, the distance between *m*_*i*_ and *m*_*j*_ is defined as:

5 $$d({m_i},{m_j})=\sqrt {\frac{1}{2}\left\| {{m_i} - {m_j}} \right\|}$$

Where ||·|| denotes the Euclidean norm. A larger value of $\:d\left({m}_{1},{m}_{2}\right)$ indicates a greater degree of conflict and lower consistency between the two trust components.

6) Trust aggregation: This is a stage that consolidates known information to assess the final trust value. Common evaluation methods include the *Dempster-Shafer* synthesis rule^23^, Bayesian inference, fuzzy logic and subjective logic. Both Bayesian inference and the *Dempster-Shafer* synthesis rule are probability-based computational method. However, Bayesian inference relies on prior probabilities to calculate posterior probabilities, which is difficult to determine in practical applications. The *Dempster-Shafer* synthesis rule focuses more on handling uncertainty and ambiguity, making it more suitable for evidence aggregation. Although fuzzy logic can also aggregate information across multiple dimensions in situations of incomplete data, the number of fuzzy rules may quickly increase in trust aggregation scenarios, making the system difficult to manage and maintain. Additionally, constructing a fuzzy logic system typically requires extensive expert knowledge, which can introduce subjectivity and bias. Similarly, subjective logic provides a more general framework for representing and combining subjective opinions. While *Dempster-Shafer* synthesis rule is more focused on combining evidence from multiple sources. Therefore, the *Dempster-Shafer* synthesis rule is the best choice for the problems considered in this paper.

Assume there are *n* evidence items {*m*^1^, *m*^2^, …, *m*^*n*^}. The probability function of event *A* given each piece of evidence can be expressed as $\{ {m^1}(A),{m^2}(A),...,{m^n}(A)\}$. By using the *Dempster-Shafer* synthesis rule, the event probability functions of these *n* evidences can be synthesized. The final event probability function of event $\:A$ is calculated as $\:{m}^{*}\left(A\right)(A\subset\:P({\Omega\:}\left)\right)$ with the following computational procedure Eq. (6). In Eq. (6), $\:K$ is the parameter to measure the degree of conflict between individual evidences. The $\:X$ and $\:Y$ are subsets in $\:P\left(\varOmega\:\right)$.

6 $$\left\{ \begin{gathered} {m^*}(A)={m^1}(A) \oplus {m^2}(A) \oplus ... \oplus {m^n}(A) \hfill \\ \;\;\;{\kern 1pt} \;\;\;{\kern 1pt} \;\;\;{\kern 1pt} =\frac{1}{{1 - K}}\sum\limits_{{{X_1} \cap {X_2} \cap ... \cap {X_n}=A}} {{m^1}({X_1}){m^2}({X_2}) \cdots {m^n}({X_n})} \hfill \\ K=\sum\limits_{{{X_1} \cap {X_2} \cap ... \cap {X_n}=\emptyset }} {{m^1}({X_1}){m^2}({X_2}) \cdots {m^n}({X_n})} \hfill \\ \end{gathered} \right.$$

The *Dempster-Shafer* synthesis rule is extensively used in our model, including the calculations of direct trust, recommended trust, and aggregated trust (see the following sections). However, our model differs from other DS-based models in several key aspects. First, our module is integrated with differential privacy to protect vehicle identities in DITDP, a feature not commonly found in other DS-based models. Second, DITDP employs a dynamic trust evaluation process that adapts to changing vehicle behavior, whereas many DS-based models use static trust assessments.

### Vehicle direct trust

There is a certain degree of history interaction behavior for intra-domain vehicles. We construct quantitative evidence through the history interaction behavior. Thus, we combine evidences $\:E\left\{{e}_{1},\:{e}_{2}\right\}$ to quantify the direct trust value $\:D{T}_{ij}$. It refers to the vehicle’s subjective judgment and evaluation of the target vehicle.

7 $$D{T_{ij}}={(m_{{ij}}^{d}\left( {{A_1}} \right),m_{{ij}}^{d}\left( {{A_2}} \right),m_{{ij}}^{d}\left( {{A_3}} \right))^T}$$

First, the evidence $\:{e}_{1}$ provides a basis for quantifying direct trust through the statistical calculation of the proportion of reliable and false packets. Suppose that the number of reliable packets provided by target vehicle is $\:{N}_{health}$, the number of false packets is $\:{N}_{false}$, and the total number of all delivered packets is $\:{N}_{all}$. Then we set the percentage of reliable packets over all delivered packets to be $\:{a}_{1}$, the percentage of false packets to be $\:{b}_{1}$, and the percentage of uncertain packets to be $\:{c}_{1}$. Thus, they can be calculated by Eq. (8).

8 $$\left\{ \begin{gathered} {N_{all}}>{N_{false}}+{N_{health}} \hfill \\ {a_1}=\frac{{{N_{health}}}}{{{N_{all}}}},{b_1}=\frac{{{N_{false}}}}{{{N_{all}}}} \hfill \\ a{}_{1}+{b_1}+{c_1}=1 \hfill \\ \end{gathered} \right.$$

For evidence $\:{e}_{2}$, we define the $\:{T}_{\mathrm{t}\mathrm{h}\mathrm{r}\mathrm{e}\mathrm{d}}$ as the packet forwarding time threshold. Based on the $\:{T}_{\mathrm{t}\mathrm{h}\mathrm{r}\mathrm{e}\mathrm{d}}$, we can get three types of data transmission behaviors: (a) timely response, which represents packet response delay less than $\:{T}_{\mathrm{t}\mathrm{h}\mathrm{r}\mathrm{e}\mathrm{d}}$; (b) delayed response, which represents packet response delay greater than $\:{T}_{\mathrm{t}\mathrm{h}\mathrm{r}\mathrm{e}\mathrm{d}}$; (c) uncertain response, which represents packet response delay equal to $\:{T}_{\mathrm{t}\mathrm{h}\mathrm{r}\mathrm{e}\mathrm{d}}$, or the case where response time cannot be measured. While packet time delay can be influenced by factors beyond the sender’s control, such as network congestion and receiver processing capabilities, we mitigate these effects by: (1) Averaging packet time delay over a longer time window to reduce the impact of short-term fluctuations; (2) Considering packet time delay in conjunction with other evidence factors (such as message reliability) to provide a more holistic assessment of trust; (3) Normalizing the delay of a vehicle in the local area and subtracting it from its sending packets. Therefore, it can be used to reduce the impact of the communication structure. Here, the $\:{Q}_{timely}$ denotes the number of timely response packets, and $\:{Q}_{delay}$ denotes the number of delayed response packets. The $\:{a}_{2}$, $\:{b}_{2}$, $\:{c}_{2}$ are the proportion of different packets (timely response packet, delayed response packet, uncertain response packet), respectively. Thus, they can be calculated by Eq. (9).

9 $$\left\{ \begin{gathered} {Q_{all}}>{Q_{timely}}+{Q_{delay}} \hfill \\ {a_2}=\frac{{{Q_{timely}}}}{{{Q_{all}}}},{b_2}=\frac{{{Q_{delay}}}}{{{Q_{all}}}} \hfill \\ {a_2}+{b_2}+{c_2}=1 \hfill \\ \end{gathered} \right.$$

Therefore, the direct trust value $\:D{T}_{ij}$ of vehicle $\:{V}_{i}$ to the target vehicle $\:{V}_{j}$ through the evidence set $\:E\left\{{e}_{1},\:{e}_{2}\right\}$ can be expressed as Eq. (10).

10 $$\begin{gathered} D{T_{ij}}={(m_{{ij}}^{d}\left( {{A_1}} \right),m_{{ij}}^{d}\left( {{A_2}} \right),m_{{ij}}^{d}\left( {{A_3}} \right))^T} \hfill \\ \quad \quad ={({a_1} \oplus {a_2},{b_1} \oplus {b_2},{c_1} \oplus {c_2})^T} \hfill \\ \end{gathered}$$

where $$m_{{ij}}^{d}({A_1})={m^{{e_1}}}({A_1}) \oplus {m^{{e_2}}}({A_1})={a_1} \oplus {a_2}$$.

### Vehicle recommended trust

In our identity trust evaluation model, a strict vehicle trust determination strategy is adopted. When the credible component $m_{{ij}}^{d}\left( {{A_1}} \right)$ in $\:D{T}_{ij}$ is not less than the threshold $\:\tau\:$ (In the following text, it will be referred to directly as $D{T_{ij}} \geqslant \tau$), determining the trustworthiness of the target vehicle $\:{V}_{j}$ solely based on the direct trust value is one-sided. In this case, the recommended trust value $\:R{T}_{j}$ should be considered. The recommended trust value $\:R{T}_{j}$ for the target vehicle *V*_*j*_ is synthesized from the direct trust components provided by other vehicles within the domain, excluding the requesting vehicle *V*_*i*_. This ensures that $\:R{T}_{j}$ represents the collective opinion of third-party observers. Thus, $\:R{T}_{j}$ is a synthesis of the trust components of other vehicles to the target$\:\:{V}_{j}$. The $\:R{T}_{j}$ is defined in the form shown in Eq. (11).

11 $$R{T_j}={(m_{j}^{r}\left( {{A_1}} \right),m_{j}^{r}\left( {{A_2}} \right),m_{j}^{r}\left( {{A_3}} \right))^T}$$

**Fig. 3:**
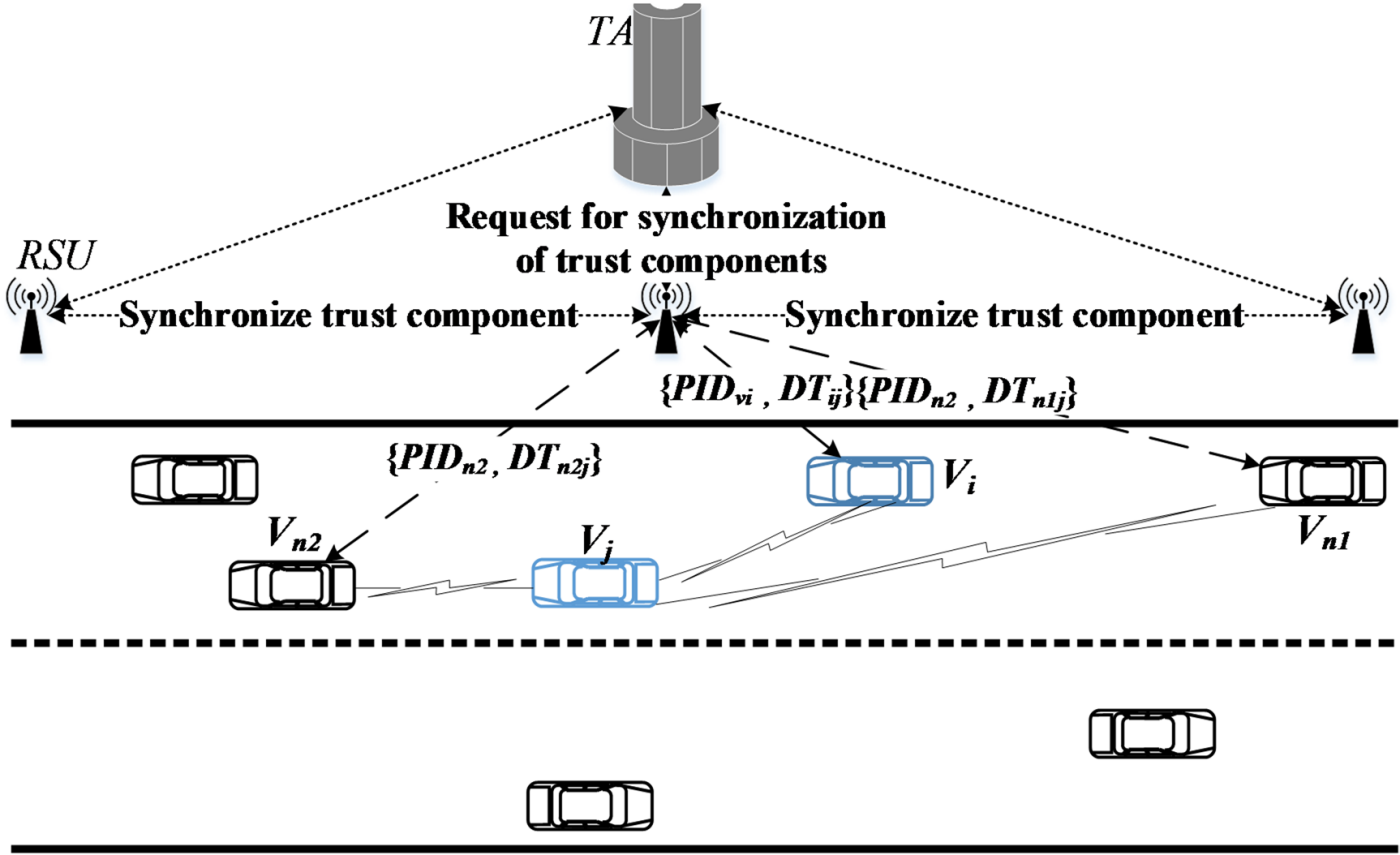
Trust component collection process.

After the vehicle completes the local direct trust update, it uploads the latest trust component to the edge trust entity RSU. The RSU undertakes the collection of all trust components and the calculation of the recommended trust. The trust component collection process can be shown in Fig. 3.

In RSU, hash is used as the data structure to store the trust evaluation results for target vehicle $\:{V}_{j}$. Its structure is shown in Table 2, with the primary key as *Key_V*_*id*_ and secondary keys as {*Value_List_DT*_*id*_, *Reco_Trust*, *Agg_Trust*, $\:{T}_{{AT}_{validity}}$}. *Key_V*_*id*_ is the unique identifier of a vehicle. *Value_List_DT*_*id*_ is a data structure of a kind of chain table to store the trust component of $\:{V}_{j}$, containing $\:\{D{T}_{1j},\:D{T}_{2j},\:\dots\:,\:D{T}_{kj}\}$. *Reco_Trust* is the recommended trust value, aggregated based on recommendations from other vehicles. *Agg_Trust* is the final trust value after combining direct and recommended trust. $\:{T}_{{AT}_{validity}}$ is the validity period of AT.

**Table 2:** Vehicle trust data storage table in RSU.

Key_V_id_	Value_List_DT_id_	Reco_Trust	Agg_Trust	$\:{\boldsymbol{T}}_{\boldsymbol{A}{\boldsymbol{T}}_{\boldsymbol{v}\boldsymbol{a}\boldsymbol{l}\boldsymbol{i}\boldsymbol{d}\boldsymbol{i}\boldsymbol{t}\boldsymbol{y}}}$
*V*_*j*_	*List*_*j*_={*DT*_*aj*_, *DT*_*bj*_ }	*RT*_*id*_	*AT*_*id*_	*T*_*j*_

When the recommended trust value $\:R{T}_{j}$ of the target vehicle $\:{V}_{j}$ needs to be calculated, the RSU can either retrieve all the trust components $\:D{T}_{ij}$ in the local database or synchronize the trust components stored in other RSUs through trusted authoritative institutions (TA).

Then, RSU further computes the recommended trust value $\:R{T}_{j}$ for $\:{V}_{j}$ by all trust components of $\:{V}_{j}$ based on Eq. (12).

12 $${\text{ }}\left\{ \begin{gathered} m_{j}^{r}(A)=m_{{1j}}^{d}(A) \oplus m_{{2j}}^{d}(A) \oplus ... \oplus m_{{kj}}^{d}(A) \hfill \\ R{T_j}={(m_{j}^{r}\left( {{A_1}} \right),m_{j}^{r}\left( {{A_2}} \right),m_{j}^{r}\left( {{A_3}} \right))^T} \hfill \\ \end{gathered} \right.$$

### Vehicle aggregated trust

The aggregated trust is the goal of vehicle trust evaluation. It is composed of direct trust and recommended trust through a certain algorithm. In this paper, the direct trust $\:D{T}_{ij}$ and the recommended trust $\:R{T}_{j}$ are synthesized by the *Dempster* synthesis rule. And finally, the aggregated trust $\:A{T}_{j}$ for the target vehicle $\:{V}_{j}$ is obtained, which satisfies the equation shown in Eq. (13).

13 $$\begin{gathered} A{T_j}={(m_{j}^{a}({A_1}),m_{j}^{a}({A_2}),m_{j}^{a}({A_3}))^T} \hfill \\ m_{j}^{a}(A)=m_{j}^{r}(A) \oplus m_{{ij}}^{d}(A) \hfill \\ m_{j}^{r}(A) \subset R{T_j},m_{{ij}}^{d}(A) \subset DT_{{ij}}^{{}} \hfill \\ \end{gathered}$$

Aggregated trust *AT*_*j*_ serves as the ultimate objective of vehicle trust evaluation. It is synthesized by integrating the collective recommended trust *RT*_*j*_ (from other vehicles) with the requesting vehicle’s own direct trust *DT*_*ij*_ toward the target vehicle *V*_*j*_. This process effectively combines social recommendations with personal experience.

If the target vehicle $\:{V}_{j}$ has no interaction history with other vehicles, the trust component of vehicle $\:{V}_{j}$ cannot be obtained. At this point, the direct trust $\:D{T}_{ij}$ of vehicle $\:{V}_{i}$ to target vehicle $\:{V}_{j}$ can be used as the aggregated trust $\:A{T}_{j}$ of $\:{V}_{j}$. Let $\:A{T}_{j}^{f}$ represent the $\:{m}_{j}^{a}\left({A}_{1}\right)$ component of the aggregated trust vector $\:A{T}_{j}$, which is the credibility degree of the vehicle. $\:A{T}_{j}^{f}$ is the trust value that is eventually stored in the local database by the RSU, which is the final basis for the vehicle to judge the credibility of the evaluated object. The RSU dynamically uploads the latest aggregated trust value of the vehicle to TA based on the $\:{T}_{jA{T}_{validity}}$value, in order to synchronize the data of all RSUs within TA’s management range.

14 $$\begin{gathered} A{T_j}={(m_{j}^{a}\left( {{A_1}} \right),m_{j}^{a}\left( {{A_2}} \right),m_{j}^{a}({A_3}))^T} \hfill \\ \;\;\;{\kern 1pt} \;\;\;{\kern 1pt} ={(AT_{1}^{f},AT_{2}^{f},...,AT_{j}^{f})^T} \hfill \\ \end{gathered}$$

## Algorithm design

This section describes DITDP algorithm in detail. It realizes the dynamic assessment and update of vehicle trust, resolving the conflict between vehicle trust value and identity privacy security.

### DITDP design

The DITDP algorithm is shown in *Algorithm 1*. The vehicle obtains the trust value of the target vehicle from the trust storage center obtained by the RSU. If it is found and valid, it is returned directly. If it cannot be found, the RSU calls the dynamic trust evaluation module to generate an aggregated trust value for the target vehicle. Vehicles first need to evaluate direct trust based on the historical behavior of the target vehicle. Secondly, they must also consider trust information from other vehicles to compute the recommended trust. Thirdly, direct trust and recommended trust are synthesized into aggregated trust using the *Dempster-Shafer* synthesis rule. By performing an exponentially weighted moving average of the aggregated trust, it achieves control of the influence of past aggregated trust values on the current aggregated trust values. Thus, the dynamic trust evaluation module can recalculate the aggregated trust value to ensure the timeliness and availability of the trust value.

Next, the identity privacy protection module is applied to the aggregated trust value, removing sensitive information and private data from the trust value. This step employs a differential privacy mechanism, using the *Staircase* mechanism^24^ to add noise and generate perturbed trust values. The probability density function of the *Staircase* perturbation mechanism shows a step shape, which has a smoother curve and is more dispersed in distribution. This guarantees that vehicles cannot obtain private information about others based on the perturbed trust values. Finally, the aggregated trust values are stored in the trust storage center and returned to the requested vehicle.

In this paper, the *Staircase* mechanism is chosen over Laplace and Gaussian noise mechanisms due to its superior privacy-utility trade-off. While Laplace and Gaussian mechanisms provide strong differential privacy guarantees, they can introduce significant distortion to the trust values, potentially impacting the accuracy of trust decisions. The *Staircase* mechanism, on the other hand, introduces smaller perturbations while satisfying the same cost function and *ε-DP* protection level. It offers more fine-grained control over the perturbation, allowing us to achieve a similar level of privacy with less distortion, thereby providing better utility. This is especially important in vehicular networks, where accurate trust values are crucial for safety-critical applications. Thus, the Staircase mechanism can be employed to perturb the trust set, ensuring differential privacy protection for the queried vehicle trust values while maintaining the availability of the vehicle trust value data.

**Algorithm 1:**
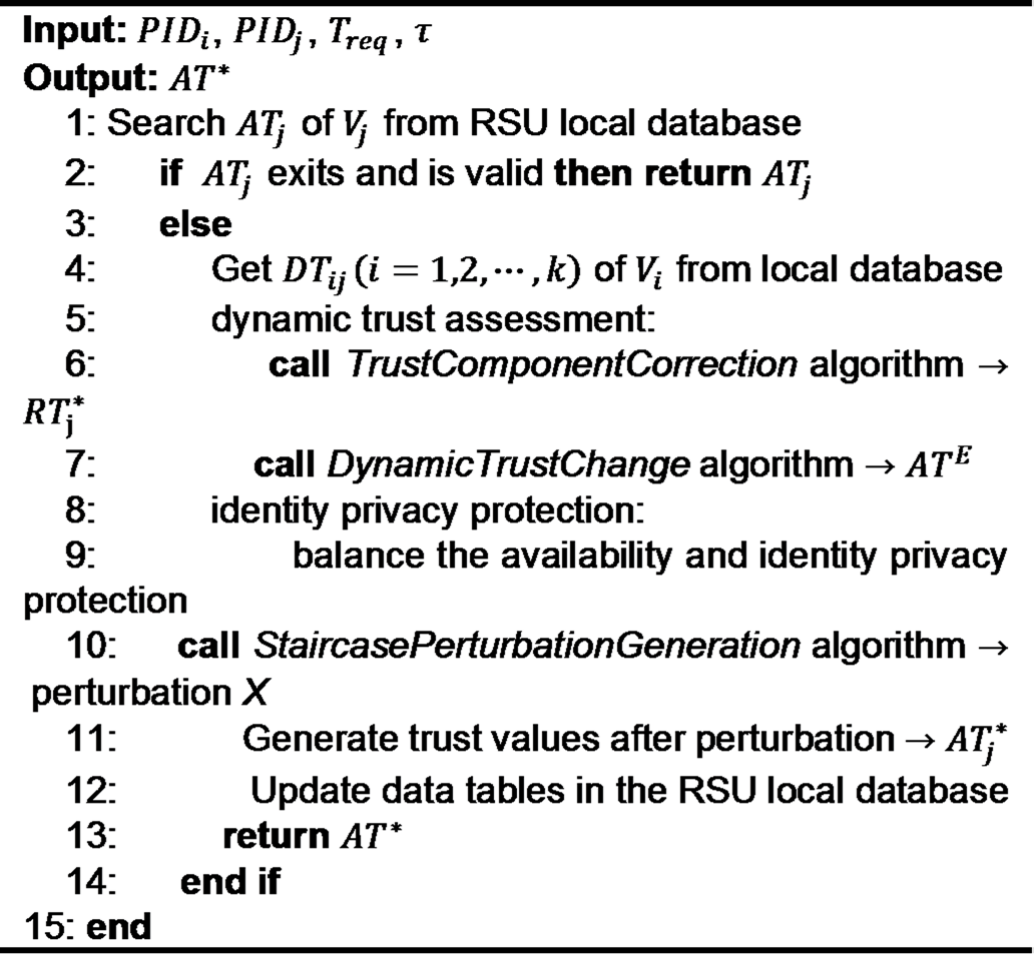
DITDP Algorithm.

### Dynamic trust assessment module

**Fig. 4:**
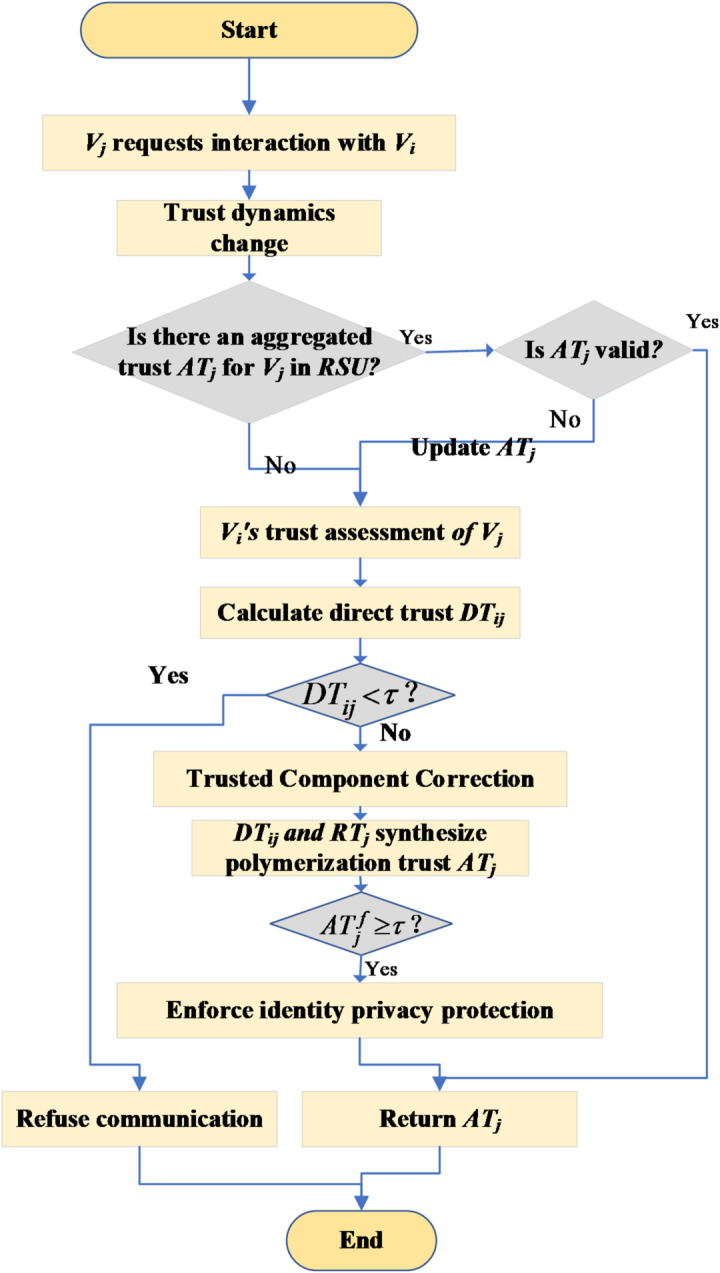
Dynamic trust assessment.

When the vehicle $\:{V}_{i}$ requests to communicate with vehicle $\:{V}_{j}$, the dynamic trust assessment is shown in Fig. 4. First, it needs to determine the trust threshold $\:\tau\:$ that it can accept. If the trust value obtained from RSU is not less than $\:\tau\:$, $\:{V}_{j}$ is considered to be trustworthy and able to interact with it for information. Otherwise, the evaluated vehicle is considered untrustworthy and corresponding measures are taken to isolate the communication and avoid the transmission of erroneous information in IoV. Then, $\:{V}_{i}$ sends a query packet carrying the target vehicle $\:{V}_{j}$‘s pseudonym identifier $\:PI{D}_{j}$ to the RSU to query the $\:A{T}_{j}$ of $\:{V}_{j}$. After RSU receives the query request packet $\:\left\{PI{D}_{i},\:PI{D}_{j},\:{T}_{req},\tau\:\right\}$ from vehicle $\:{V}_{i}$, it will resolve the vehicle genuine identity $\:{V}_{id}$ according to $\:PI{D}_{j}$. Then RSU searches whether the $\:A{T}_{j}$ exists from the local database according to $\:{V}_{id}$. There are three cases need to update the trust value: a) There is not the $\:A{T}_{j}$ in trust storage; b) There is the $\:A{T}_{j}<\tau\:$. It means that $\:{V}_{j\:}$ is considered untrustworthy; c) The $\:A{T}_{j}$ is not updated in a periodic time $\:{T}_{{jAT}_{validity}}$.

If the current time does not exceed the last update time $\:{T}_{{jAT}_{validity}}$ and there is the $\:A{T}_{j}>\tau\:$ for $\:{V}_{j}$, the *DITDP* directly outputs the aggregated trust $\:A{T}_{j}$. Otherwise, the aggregated trust $\:A{T}_{j}$ will be updated. First, the component $\:A{T}_{j}^{f}$ of the aggregated trust $\:A{T}_{j}$ is changed to $\:A{T}_{j}^{E}$ through the trust component correction and dynamic trust change by the module of dynamic trust assessment. Then, *DITDP* performs the module of identity privacy protection. And the final publicly available trust value $\:A{T}^{*}$ is obtained.

1) Trust Component Correction.

As show in Fig. 4, the recommended trust is derived from the independent evaluations of multiple entities regarding the target vehicle. However, these evaluations (i.e., trust components) are not always truthful and reliable. Malicious vehicles may provide false evaluations to elevate their accomplices or discredit honest vehicles. Even well-intentioned vehicles may present biased evaluations due to limitations in their observational perspectives and interaction histories. Therefore, we propose a trust component correction mechanism. The core idea is to transform the weight allocation process into a measurement of consistency among trust components. If a trust component aligns with the views of the majority of other components, its reliability is considered high and should be assigned a higher weight; conversely, if a component significantly conflicts with the group consensus, its reliability is questionable and should be assigned a lower weight to mitigate its negative impact.

Suppose the set of direct trusts of $\:{V}_{j}$ is $\:\{D{T}_{1j},\:\dots\:,\:D{T}_{kj}\}$. Through Eq. (3), RSU can calculate the conflict degree $\:{C}_{ab}$ between any two direct trusts $\:a=D{T}_{aj}$ and $\:b=D{T}_{bj}\left(a,\:b\in\:\:\left[1,\:k\right]\right)$, as shown in Eq. (15).

15 $$\begin{gathered} {\text{ }}D=\left[ {\begin{array}{*{20}{c}} {{d_{11}}}&{{d_{12}}}&{{d_{13}}} \\ {{d_{21}}}&{{d_{22}}}&{{d_{23}}} \\ {{d_{31}}}&{{d_{32}}}&{{d_{33}}} \end{array}} \right] \hfill \\ {C_{ab}}=\sqrt {\frac{1}{2}{{(D{T_{aj}} - D{T_{bj}})}^T}D(D{T_{aj}} - D{T_{bj}})} \hfill \\ \end{gathered}$$

First, the comprehensive similarity of all other direct trusts to $\:D{T}_{aj}$ is defined as $\:{\alpha\:}_{a}^{j}$. It can be calculated as shown in Eq. (16).

16 $$\alpha _{a}^{j}=k - 1 - \sum\limits_{{i=1,b \ne a}}^{k} {{C_{ab}}}$$

Then, we set the sum of similarity of $\:k$ direct trusts as $\:{\alpha\:}_{all}^{j}$. We have $\:{\alpha\:}_{all}^{j}={\sum\:}_{i=1}^{k}{\alpha\:}_{i}^{j}$. The $\:{\alpha\:}_{all}^{j}$ is used as the criterion for assigning weights. If a direct trust accounts for a larger proportion of $\:{\alpha\:}_{all}^{j}$, the weight assigned to it should be larger. In turn, the weight assigned should be smaller. We define the weight factor to the $\:D{T}_{xj}$ as $\:{{\upvarpi\:}}_{x}$. Thus, the set of corrected direct trusts can be obtained by adding weight factors to each trust component, as shown in Eq. (17).

17 $$\begin{gathered} \left[ {\begin{array}{*{20}{c}} {{\varpi _1}}&0& \cdots &0 \\ 0&{{\varpi _2}}& \cdots &0 \\ \vdots & \vdots & \ddots & \vdots \\ 0&0& \cdots &{{\varpi _k}} \end{array}} \right]\;\;\;{\kern 1pt} \times \;\;\;{\kern 1pt} \left[ \begin{gathered} D{T_{1j}}^{T} \hfill \\ D{T_{2j}}^{T} \hfill \\ ... \hfill \\ D{T_{kj}}^{T} \hfill \\ \end{gathered} \right]\;\;\;{\kern 1pt} =\;\;\;{\kern 1pt} \left[ \begin{gathered} {\varpi _1}D{T_{1j}}^{T} \hfill \\ {\varpi _2}D{T_{2j}}^{T} \hfill \\ ... \hfill \\ {\varpi _k}D{T_{kj}}^{T} \hfill \\ \end{gathered} \right] \hfill \\ \;\;\;{\kern 1pt} =\;\;\;{\kern 1pt} {\left[ \begin{gathered} {\varpi _1} \cdot m_{{1j}}^{d}\left( {{A_1}} \right)\mathop ,\limits^{{}} {\varpi _1} \cdot m_{{1j}}^{d}\left( {{A_2}} \right)\mathop ,\limits^{{}} {\varpi _1} \cdot m_{{1j}}^{d}\left( {{A_3}} \right) \hfill \\ {\varpi _2} \cdot m_{{2j}}^{d}\left( {{A_1}} \right)\mathop ,\limits^{{}} {\varpi _2} \cdot m_{{2j}}^{d}\left( {{A_2}} \right)\mathop ,\limits^{{}} {\varpi _2} \cdot m_{{2j}}^{d}\left( {{A_3}} \right) \hfill \\ ... \hfill \\ {\varpi _k} \cdot m_{{kj}}^{d}\left( {{A_1}} \right)\mathop ,\limits^{{}} {\varpi _k} \cdot m_{{kj}}^{d}\left( {{A_2}} \right)\mathop ,\limits^{{}} {\varpi _k} \cdot m_{{kj}}^{d}\left( {{A_3}} \right) \hfill \\ \end{gathered} \right]_{}} \hfill \\ \end{gathered}$$

Finally, the recommended trust value $\:R{T}_{j}^{*}$ can be calculated by Eq. (18).

18 $${\text{ }}\left\{ \begin{gathered} m_{j}^{{r*}}(A)=m_{{1j}}^{c}(A) \oplus m_{{2j}}^{c}(A) \oplus \ldots \oplus m_{{kj}}^{c}(A) \hfill \\ m_{{kj}}^{c}(A)={\varpi _k} \times m_{{kj}}^{d}\left( A \right) \hfill \\ RT_{j}^{*}={(m_{j}^{{r*}}\left( {{A_1}} \right),m_{j}^{{r*}}\left( {{A_2}} \right),m_{j}^{{r*}}\left( {{A_3}} \right))^T} \hfill \\ \end{gathered} \right.$$

Now we can give *Algorithm* 2 to implement the trust component correction. The time complexity of *Algorithm 2* is $\:O\left({k}^{2}\right)$. The degree of conflict between direct trusts is measured by calculating the trust distance between each trust component. The false recommendations provided by a single or a few malicious vehicles will generate a high degree of conflict with the genuine recommendations from honest vehicles. Therefore, during the weighting process, the weights of these false recommendations will be significantly reduced, greatly diminishing their impact on the final recommended trust value. If a group of malicious vehicles colludes, their internal evaluations will be highly consistent. But there will be a high conflict with the evaluations from the group of honest vehicles. At this point, two “opinion camps” will form within the system. Since the number of vehicles in the malicious camp is usually smaller than that in the honest camp, their overall similarity will still be low, leading to a lower weight being assigned, which effectively protects the recommended trust.

However, it is undeniable that the trust component correction mechanism is based on a core assumption: the majority of vehicles within the domain are honest and provide relatively accurate trust evaluations.​ When facing systematic large-scale collusion attacks (e.g., where the number of malicious vehicles exceeds that of honest ones) or Sybil attacks, the reports from honest nodes may instead become statistical “outliers” and be suppressed, leading to the failure of the correction mechanism. This is a known theoretical limitation of trust models based on consistency checks. In the context of this study, which focuses on intra-domain vehicles registered with a trusted authority, launching such large-scale attacks is considerably more difficult. Therefore, this assumption holds practical rationality.

**Algorithm 2:**
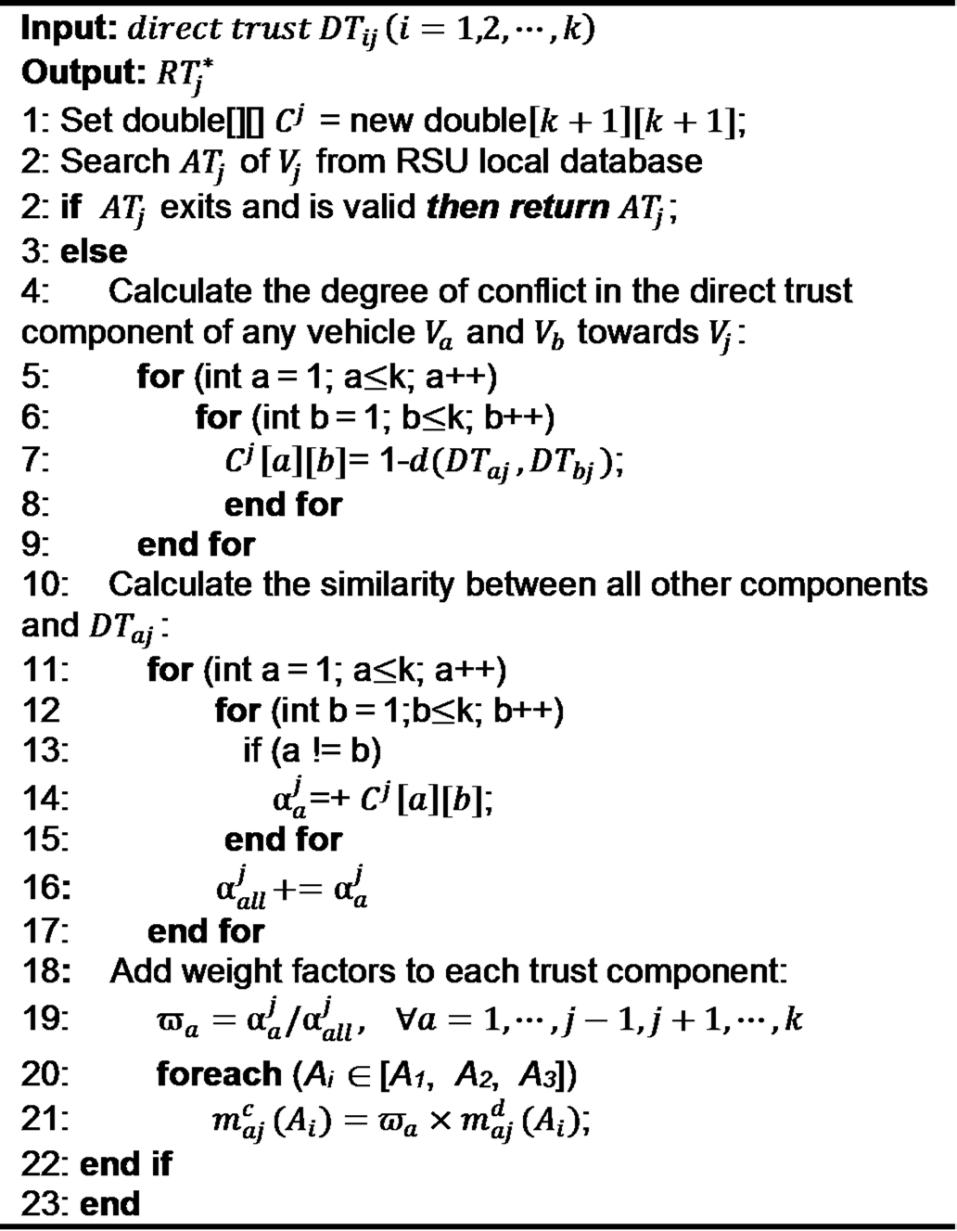
Trust_Component_Correction.

2) Trust Dynamics Change.

In the highly dynamic environment of the IoV, the trust status of vehicles is not static. A trusted vehicle may become unreliable due to attacks or abnormal behavior; conversely, a vehicle previously labeled as malicious should have the opportunity to restore its reputation after addressing the issues. Static trust assessment models cannot reflect this dynamism, leading to two major risks: latency, which fails to respond promptly to the actual changes in vehicle behavior, resulting in ineffective trust assessments; and vulnerability, which is susceptible to short-term masquerade attacks (where malicious vehicles exhibit good behavior briefly before key interactions to boost their trust values). To address these issues, we have designed a trust dynamics change mechanism aimed at achieving real-time, accurate, and smooth tracking and updating of vehicle trust.

The trust dynamics change can recalculate the trust value of related vehicles with periodic time. Assuming that the update cycle of the aggregated trust value $\:A{T}_{j}$ is $\:{T}_{j}$. When the $\:A{T}_{j}$ is outdated ($\:{t}_{cur}-{t}_{last\_update}>{T}_{j}$), it will be updated again starting from the direct trust. Assume that the vehicle has three sequentially delayed time points $\:\left\{{t}_{0},\:{t}_{1},\:{t}_{2}\right\}$. At time $\:{t}_{0}$, vehicle $\:{V}_{j}$ does not have any historical interaction with surrounding vehicles. It does not quantify the historical data interaction by the two evidence behaviors $\:E\left\{{e}_{1},\:{e}_{2}\right\}$. Therefore, at time $\:{t}_{0}$, the direct trust vector of vehicle $\:{V}_{i}$ to target vehicle $\:{V}_{j}$ can be expressed as $\:D{T}_{ij}\left({t}_{0}\right)={\left(0,\:0,\:1\right)}^{T}$; When $\:t={t}_{1}$, if the direct trusts for vehicles $\:{V}_{j}$ are stored in RSU, the recommended trust value $\:R{T}_{j}$ is calculated by *Algorithm 2*. And it obtains$\:\:A{T}_{j}\left({t}_{1}\right)$ at the moment of $\:{t}_{1}$; When $\:t={t}_{2}$, suppose that $\:A{T}_{j}\left({t}_{1}\right)$ is outdated. Then, the aggregated trust will be recalculated at time $\:{t}_{2}$. It takes the trust component whose time is between $\:{t}_{1}$ and $\:{t}_{2}$ and performs a new round of aggregation to obtain $\:A{T}_{j}\left({t}_{2}\right)$.

The trust value changes too much will lead to a large deviation, especially for malicious vehicles to change their own trust value drastically. The aggregated trust value is smoothed using an exponentially weighted moving average (EWMA) to reduce the impact of sudden changes in trust. The calculation process is shown in Eq. (19).

19 $$AT_{j}^{E}({t_{cur}})=(1 - \alpha ) \times A{T_j}({t_{cur}})+\alpha AT_{j}^{E}({t_{last}})$$

Where $\:A{T}_{j}\left({t}_{cur}\right)$ is the true trust value at moment $\:{t}_{cur}$. $\:A{T}_{j}^{E}\left({t}_{cur}\right)$ is the smoothed trust value at moment $\:{t}_{cur}$. The $\:{\upalpha\:}$ represents the smoothing factor, which usually takes the value of $\:0<\:{\upalpha\:}\:<1$. It represents the weight given to the past trust value. When$\:\:{\upalpha\:}$ is larger, the greater the effect of past trust values on current trust values, the stronger the smoothing effect. Conversely, when $\:{\upalpha\:}$ is smaller, the greater the influence of the current trust value on the smoothed trust value, the weaker the smoothing effect. The initial smoothing trust value can be set as $\:A{T}_{j}^{E}\left({t}_{0}\right)=A{T}_{j}\left({t}_{0}\right)$. After finishing updating the latest aggregation trust, the validity period of the aggregation trust needs to be dynamically adjusted. And the $\:A{T}_{j}^{E}$ that achieves a high trust value should have a long validity period. The closer the $\:A{T}_{j}$ trust value is to $\:\tau\:$, the shorter the validity period $\:{T}_{j}$ is. The trustworthiness of vehicle needs to be evaluated more frequently. Therefore, we design an adaptive update scheme for the aggregated trust $\:A{T}_{j}\left({t}_{cur}\right)$to dynamically update the validity period $\:{T}_{j}$ of the trust value. The validity period of $\:A{T}_{j}\left({t}_{cur}\right)$ is updated by Eq. (20).

20 $$\left\{ \begin{gathered} {T_j}=\hbox{max} ({e^{ - \lambda t}},{T_{\hbox{min} }}) \hfill \\ \lambda =a(1 - {e^{ - b \times d}}) \hfill \\ \end{gathered} \right.$$

Where $\:a$ and $\:b$ are the parameters of the decay function, and $\:d=A{T}_{j}^{E}\left({t}_{cur}\right)-\tau\:$. When $\:d$ is small (that is, the trust value is close to the threshold), the decay coefficient $\:{\uplambda\:}$ is larger and the period of validity is shorter. When *d* is large (that is, the trust value is far from the threshold), the decay coefficient $\:\lambda\:$ is smaller and the period of validity is longer. $\:{T}_{min}$ is the shortest period of trust validity, and $\:{T}_{j}\:$should be considered as $\:{T}_{min}$ when its appearance is less than $\:{T}_{min}$. The $\:t={t}_{cur}{t}_{last\_update}$ denotes the interval between the current time and the last update time.

With the above principle, we implement *Algorithm* 3 to periodically update the aggregated trust $\:A{T}_{j}$ and the trust validity period $\:{T}_{j}$. The input of *Algorithm* 3 is the trust component of the evaluated vehicle $\:D{T}_{ij}\left(i=\mathrm{1,2},\cdots\:,k\right)$ whose time is between $\:{t}_{last\_update}$ and $\:{t}_{cur}$, and the last updated final trust value $\:A{T}_{j}^{E}\left({t}_{last}\right)$. The output of the *Algorithm* 3 is the current new trust value $\:A{T}_{j}^{E}\left({t}_{cur}\right)$ and the corresponding validity period $\:{T}_{cur}$. It introduces “inertia.” A malicious vehicle cannot cause its trust value to skyrocket instantly through a single or short-term act of “pretending to behave well.” Similarly, it cannot quickly devalue a reputable vehicle through brief negative behavior. The changes in trust values are gradual, allowing the system to observe behavior patterns over a longer period, effectively filtering out short-term, sudden malicious manipulations.

**Algorithm 3:**
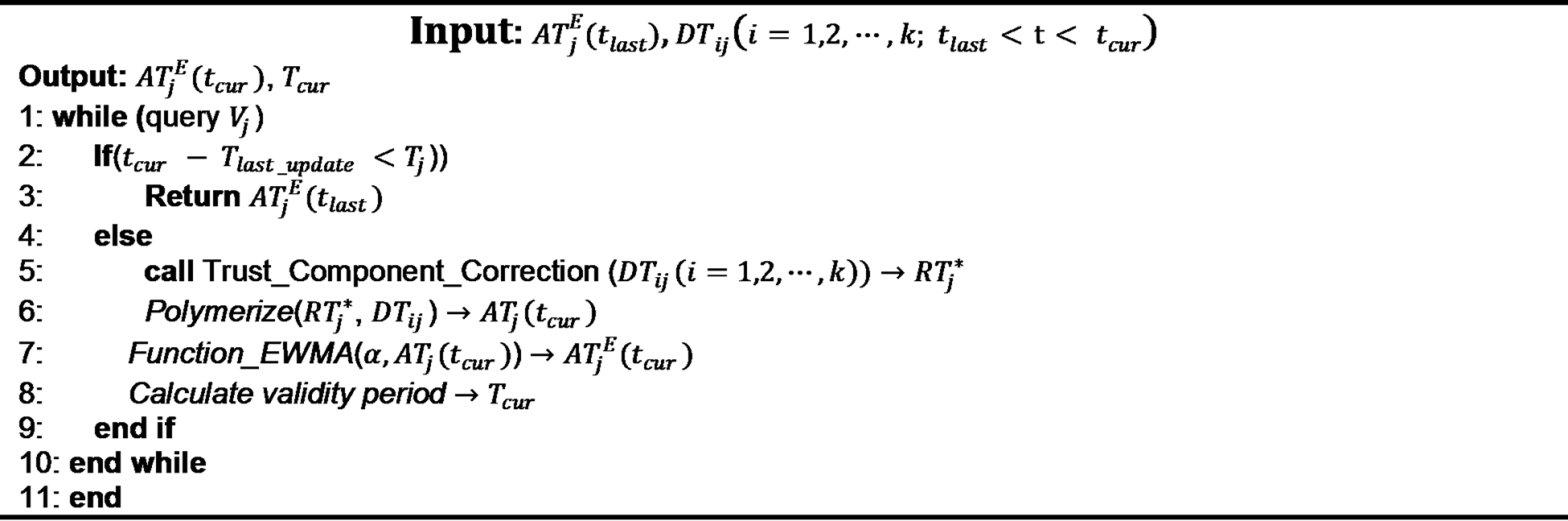
Trust_Dynamics_Change.

### Identity privacy protection module

**Fig. 5:**
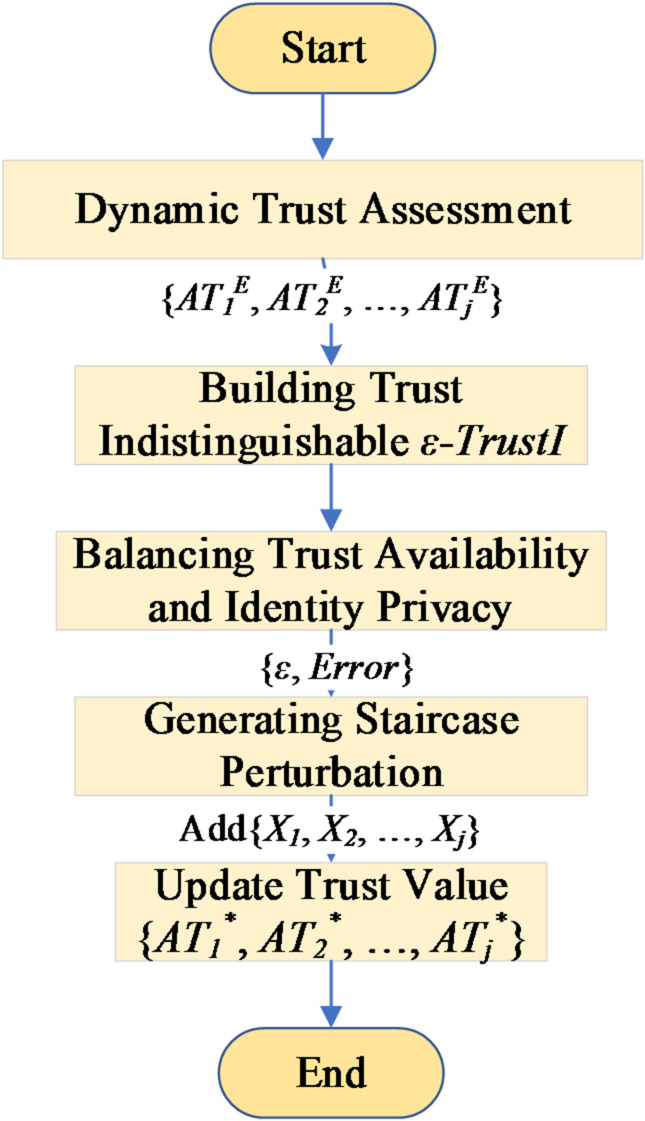
Vehicle identity privacy protection.

In the *DITDP algorithm*, vehicle identity privacy protection is implemented to achieve indistinguishability of the aggregated trust values. It provides identity privacy protection for vehicles while ensuring trust availability. The operation procedure for identity privacy protection ($\:A{T}_{j}^{E}\to\:\:A{T}_{j}^{*}$) is shown in Fig. 5. This process involves three parts: the establishing indistinguishable trust, balancing the trust availability and the identity privacy protection, and generating *Staircase* perturbations. Finally, it outputs the publicly announced trust value $\:A{T}_{j}^{*}$.

1) Indistinguishable trust.

Trust-Indistinguishability (*TrustI*) can be described as the inability of an attacker to distinguish the identity of a vehicle by observing the perturbed trust values multiple times.

First, we design the random perturbation mechanism *ℳ* with $\:S\to\:Z$, where $\:S=\left\{A{T}_{1}^{E},\:A{T}_{2}^{E},\:\dots\:,\:A{T}_{k}^{E}\right\}$, and $\:Z\:=\:\left\{A{T}_{1}^{*},\:A{T}_{2}^{*},\:\dots\:,\:A{T}_{k}^{*}\right\}$. It needs to be ensured that *ℳ*(*s*) and *ℳ*(*s*') are similar to some extent for any two trust values $\:{s}_{1},{s}_{2}\in\:S$. Even if the attacker obtains the output of the random perturbation mechanism ℳ, the true trust value cannot be obtained to some extent. Therefore, attackers cannot obtain their identity background information by querying the trust values of the same vehicle multiple times in succession.

Given two distributions $\:{z}_{1}=\mathcal{M}\left({s}_{1}\right)$ and $\:{z}_{2}=\mathcal{M}\left({s}_{2}\right)$ on *Z*, we define the multiplicative distance $\:{d}_{p}\left({z}_{1},{z}_{2}\right)$ and the Euclidean distance $\:L\left({s}_{1},{s}_{2}\right)$ between these two distributions^25^, as in Eq. (21).

21 $$\left\{ \begin{gathered} {d_p}({z_1},{z_2})={\sup _{z \in {\rm Z}}}\left| {\ln \frac{{{z_1}(Z)}}{{{z_2}(Z)}}} \right| \hfill \\ L({s_1},{s_2})=\sqrt {\sum\nolimits_{{i=1}}^{n} {(s_{1}^{i} - s_{2}^{i})} } \hfill \\ \end{gathered} \right.$$

Thus, for the trust indistinguishability mechanism (*ε-TrustI*) satisfies Eq. (22).

22 $${d_p}(\mathcal{M}({s_1}),\mathcal{M}({s_2})) \leqslant \varepsilon L{\mathrm{(}}{s_1},{s_2}{\mathrm{)}}$$

The *ε-TrustI* means that when there are real vehicle trusts as $\:{s}_{1}$ and $\:{s}_{2}$. They generate external perturbation trusts as $\:{z}_{1}$ and $\:{z}_{2}$ by differential privacy perturbation function. The Eq. (22) is satisfied between real and perturbation trusts.

2) Trust availability and privacy protection balance.

The *ε-TrustI* is a way to protect the privacy of the vehicle trust values. However, adding perturbations to the true trust values has an impact on the availability of trust values. Thus, we analyze the trade-off between data availability and privacy protection.

The smaller of the privacy budget $\:\epsilon\:$ means that the greater the protection of the trust value data. This leads to the worse of the trust value availability. In this paper, assume that the trust offset between the true trust value $\:s$ and the perturbed trust value $\:z$ be *Error*. The trust value availability depends on the trust loss probability$\:\:\eta\:$, which represents the probability that a perturbed trust value exceeds the $\:Error$. For vehicle trusts that are closer to the trust threshold $\:\tau\:$, there is a problem of misclassification when the perturbation is added. For example, the real trust value of the vehicle is $\:s$ and$\:\:s>\tau\:$, the perturbed trust value is $\:z$ and $\:z\:<{\uptau\:}$. The vehicle is judged to be a trusted vehicle based on the real trust value *s*. But in fact, it is judged to be a malicious vehicle based on its perturbed trust value $\:z$. Thus, the relationship between trust loss probability $\:\eta\:$ and privacy budget $\:\epsilon\:$ needs to be determined.

Let the Euclidean distance between the true trust value and the perturbed trust value be $\:L\left({s}_{1},{z}_{1}\right)=l$. The larger $\:\eta\:$ represents the higher the vehicle’s acceptance of the trust value and the larger the perturbation that can be accepted. Thus, the random mechanism *ℳ* can satisfy the maximum bound of the vehicle acceptance trust deviation as Eq. (23).

23 $$\begin{gathered} Pr(l \geqslant Error)=1 - Pr( - Error \leqslant l \leqslant Error) \hfill \\ \quad \;\;\;\quad \;\;\;\quad \;\;=1 - \int_{{ - Error}}^{{Error}} {f(x)} dx \leqslant \eta \hfill \\ \end{gathered}$$

Suppose the maximum offset trust that the vehicle can accept is $\:Erro{r}_{max}$ and the maximum probability of trust loss is $\:{\eta\:}_{max}$. Then, the trust availability of the vehicle is the lowest when $\:Erro{r}_{max}$ and $\:{\eta\:}_{max}$ are taken. And the privacy budget added at this time is the minimum value $\:{{\upepsilon\:}}_{min}$, which provides the highest degree of privacy protection. Therefore, to get the highest privacy protection for the vehicle trust value, it is necessary to get the minimum privacy budget $\:{\epsilon\:}_{min}$, which can be calculated by $\:Pr\left(l\ge\:Erro{r}_{max}\right)\le\:{{\upeta\:}}_{max}$.

3) Trust perturbation.

The *ε-TrustI* mechanism achieves the fuzzification of the true trust value by Staircase perturbation. It makes the query-derived data deviate from the true data value with a certain probability, so as to achieve the purpose of protecting data privacy.

For the *Staircase* perturbation, we achieve that the generated noisy trust value can be distributed around the true trust value ($\:{z}_{1}>{s}_{1}$ or $\:{z}_{1}<{s}_{1}$) by choosing the appropriate parameters. According to the probability density function of *ε-TrustI*, it can be achieved that the *Staircase* probability that the generated $\:{z}_{1}$ values are distributed around $\:{s}_{1}$ is the same. The closer the generated $\:{z}_{1}$ values are to $\:{s}_{1}$, the higher the *Staircase* probability. Thus, in a one-dimensional dataset, the *Staircase* probability density function is a segmented function, whose cumulative distribution function $\:cdf\left(x\right)$ can be expressed as Eq. (24).

24 $$\begin{gathered} cdf(x)=\left\{ \begin{gathered} 0.5{\text{ }}x=0 \hfill \\ 0.5+a(\gamma )x{\text{ 0}} \leqslant x<\gamma \Delta \hfill \\ 0.5+a(\gamma )[\gamma \Delta +{e^{ - \left\lfloor {\frac{{x - \gamma \Delta }}{\Delta }+1} \right\rfloor \varepsilon }}(x - (\left\lfloor {\frac{{x - \gamma \Delta }}{\Delta }} \right\rfloor +\gamma )\Delta )+\sum\limits_{{k=1}}^{{\left\lfloor {\frac{{x - \gamma \Delta }}{\Delta }} \right\rfloor }} {{e^{ - (k - 1)\varepsilon }}} {\text{] }}x \geqslant \gamma \Delta {\text{ }} \hfill \\ 1{\text{ }}x \to +\infty \hfill \\ \end{gathered} \right. \hfill \\ a(\gamma )=\frac{1}{{2\Delta (b+(1 - b)\gamma )}}=\frac{{(1+{e^{\varepsilon /2}})}}{{2\Delta (1+{e^{ - \varepsilon /2}})}} \hfill \\ \end{gathered}$$

From Eq. (24), it can be seen that $\:cdf\left(x\right)$ is monotonically increasing. Thus, it can be obtained that it is invertible and its inverse function $\:{F}^{-1}\left(x\right)$ exists. Using the random number between $\:V\in\:\left[\mathrm{0,1}\right]$ and the inverse function $\:{F}^{-1}\left(x\right)$, the specific *Staircase* perturbation can be obtained.

The pseudo-code of *Staircase_Perturbation_Generation* is shown in *Algorithm 4*. The inputs of *Algorithm 4* are privacy budget $\:\epsilon\:$, sensitivity $\:\varDelta\:$ and the trust offset $\:Error$. The output is a random perturbation $\:X$. The parameter $\:k$ is used to determine the range of the perturbation. And the larger $\:k$ is, the larger the perturbation value can be generated, which will have a greater impact on data availability. Thus, the $\:k$ can be determined from$\:\:\epsilon\:$, $\:\varDelta\:$, and $\:Error$. The positive and negative values of the added perturbation are determined by $\:R$, randomly taken from $\:\{-\mathrm{1,1}\}$ with 50% probability. $\:B$ is a binary number that determines the specific position of the perturbation in the interval. $\:U$ is used to randomly generate $\:X$ in the determined interval.

According to *Algorithm 4*, we obtain the desired trust value perturbation $\:X$. The final response to the set of perturbed trust values of the vehicle can be expressed as $\:\left\{A{T}_{1}^{*},\:A{T}_{2}^{*},\:\dots\:,\:A{T}_{j}^{*}\right\}$, where $\:A{T}_{j}^{*}A{T}_{j}^{E}+{X}_{j}$.

**Algorithm 4:**
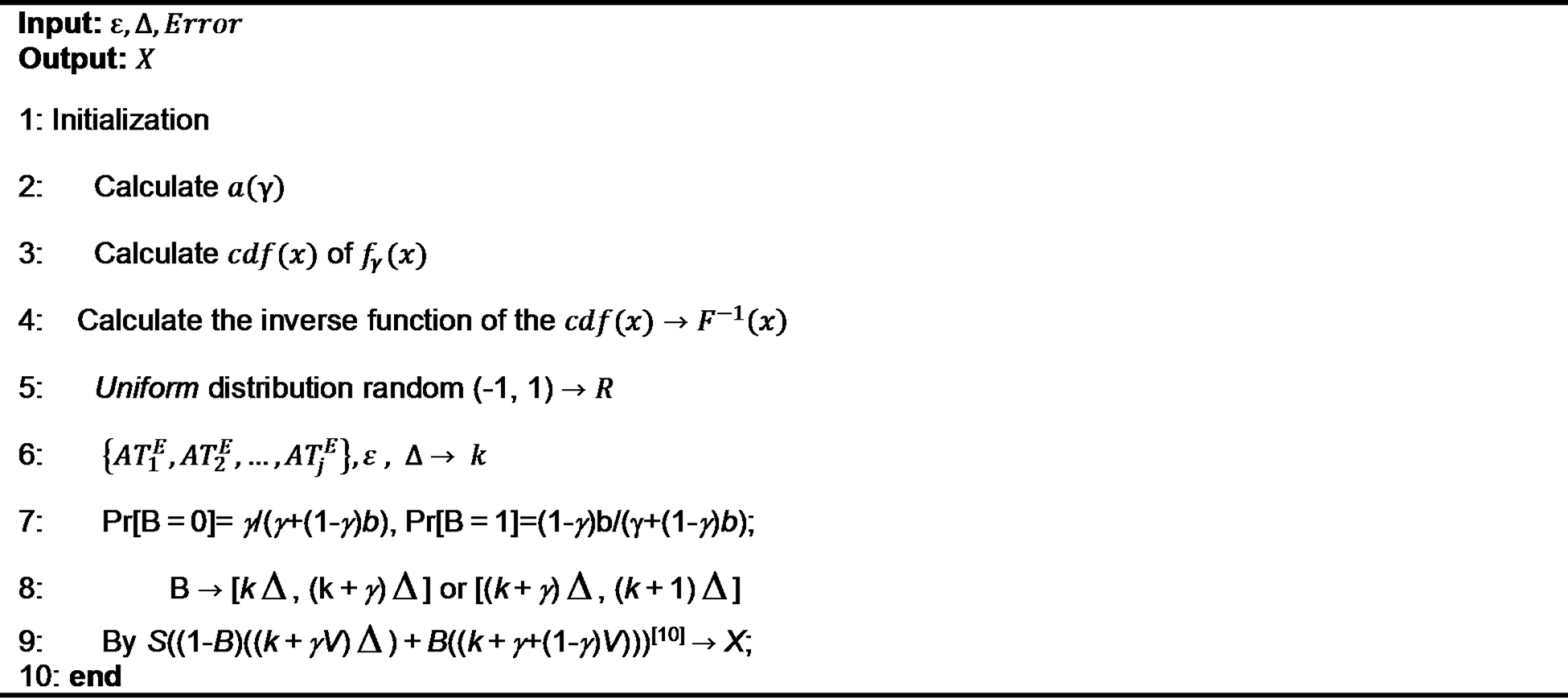
Staircase_Perturbation_Generation.

### Theoretical performance analysis of DITDP

In this section, we conduct a qualitative discussion of various performance aspects based on the design of DITDP algorithm.

1) Analysis of computational efficiency.

The computational burden of the DITDP algorithm mainly comes from three parts: trust assessment calculations, dynamic updating processes, and differential privacy perturbations.

First, vehicles locally compute direct trust values, involving statistical operations on historical interaction data (such as message ratios and response delays). These calculations are based on simple arithmetic operations (such as addition and division), with a time complexity of *O*(1), which can be considered lightweight. Second, when the RSU synthesizes recommended trust, it needs to perform trust component corrections (Algorithm 2). Algorithm 2 requires calculating the Josselme distance between all pairs of trust components, with a time complexity of *O*(*k*^2^), where *k* is the number of trust components (i.e., the number of vehicles being evaluated). In scenarios with a limited number of vehicles in the domain, the *O*(*k*^2^) overhead is acceptable. Finally, the D-S evidence synthesis rule aggregates the trust values, with a time complexity of *O*(1), making it very efficient.

In the dynamic trust assessment process (Algorithm 3), operations such as EWMA smoothing and re-calculating validity periods only involve basic mathematical computations, resulting in low computational overhead. The generation of staircase perturbations (Algorithm 4), based on differential privacy, involves probability calculations and random number generation. Its complexity depends on the inverse function calculation. Since the trust values are scalars, the overall complexity can be considered *O*(1), which does not introduce significant delays. Therefore, the computational efficiency of the DITDP algorithm is feasible in typical vehicular network scenarios.

2) Analysis of storage cost.

Memory usage is primarily concentrated on data storage at the RSU and local vehicles. The RSU uses a hash structure to store vehicle trust data, including direct trust lists, recommended trust, and aggregated trust. Each vehicle’s record contains fixed fields. Thus, the memory usage for a single vehicle is *O*(*k*), with a total usage of *O*(*n*⋅*k*), where *n* is the total number of vehicles in the domain. Trust component correction requires temporary storage for the conflict matrix (size *k*×*k*), which occupies *O*(*k*^2^) memory. However, since *k* is relatively small and can be released after computation, peak memory usage is manageable.

Vehicles only need to cache their own interaction history and local direct trust values, resulting in minimal storage overhead. The main computations rely on the RSU, which reduces the resource pressure on the vehicle side.

3) Analysis of real-time processing capabilities.

When a vehicle queries a trust value, the RSU first checks the local database. If the trust value is valid and exists, it is directly returned, resulting in a very quick response. If an update is needed, it triggers the dynamic trust assessment and privacy protection module. The RSU typically has strong computational capabilities, ensuring response times in the hundreds of milliseconds, thus meeting the real-time requirements of V2X communication in vehicular networks. Additionally, the DITDP algorithm reduces unnecessary updates through adaptive validity periods, lowering the frequency of real-time computations. Therefore, the end-to-end delay for trust value dissemination is primarily determined by the communication latency between vehicles and RSUs, the overall delay remains within acceptable bounds for real-time vehicular communication.

4) Analysis of scalability and deployment feasibility.

The scalability of DITDP is primarily determined by the computational complexity of the trust component correction and Staircase perturbation mechanisms, as well as the communication overhead for trust value dissemination. Since these mechanisms have relatively low complexity, DITDP can scale to large vehicular networks with a reasonable number of vehicles per RSU.

To further enhance scalability, DITDP can be integrated with cloud-edge infrastructures. The trust evaluation process can be offloaded to edge servers, reducing the computational burden on RSUs. Furthermore, the trust values can be stored in a distributed manner across multiple edge servers, improving the reliability and availability of trust information.

For real-world deployment, DITDP can be deployed in existing RSUs with minimal modifications. The trust evaluation process can be implemented as a software module within the RSU, and the communication protocols can be adapted to support trust value dissemination. Alternatively, DITDP can be deployed in a cloud-edge infrastructure, with RSUs acting as gateways to the cloud for trust evaluation and management.

DITDP is compatible with encrypted vehicle communications. The trust evaluation process operates on the content of the messages, which are assumed to be decrypted by the receiving vehicle using standard cryptographic techniques. The perturbed trust value is then used to assess the trustworthiness of the sender, not to modify the content of the message.

To ensure that critical safety-related messages are not negatively affected by the perturbation of trust values, DITDP can be adapted to use an adaptive privacy mechanism. For safety-critical messages, the privacy budget (*ε*) can be temporarily increased or even set to infinity, reducing or eliminating the perturbation and ensuring that the receiver has a highly accurate assessment of the sender’s trustworthiness. For non-safety-critical messages, a higher degree of privacy protection can be applied.

## Evaluation

### Simulation environment

In this paper, we will experiment with vehicle trust management through *OMNeT++*, a network simulation tool developed based on *Eclipse*. *OMNeT + +* is an open-source, component-based simulation environment that allows users to define and simulate different network topologies, protocols, and applications, with open-source libraries *INET* and *INETMANET*, etc. And it supports *C/C + +* projects. The *OMNeT + +* can implement specific network topologies and composite vehicle models by configuring .*ned* files, various behaviors of vehicle nodes by .*cc* files, specific message models by.*msg* files, and communication protocols by .*ini* files. Finally, it analyzes out files to obtain results. In the experimental simulation, we set the total number of vehicles as 50. The simulation environment and parameter configuration of this experiment are shown in Table 3.

**Table 3:** Simulation environment.

Name	Detailed configuration
CPU	12th generation Intel Core i9-12900 H
Memory	16G
Graphics card	RTX 3060
JDK	1.8
OMNeT++	6.0.1
Simulation area	3 km*5km
Number of vehicles	50
Communication Protocol	IEEE 802.11p
Channel Spectrum Bandwidth	55 kHz
Maximum packet transmission rate	2Mbps
Maximum packet transmission rate	600 m
Packet size	300Byte-400Byte
Average vehicle speed	30 km/h
Vehicle communication range (radius)	200 m
Path loss index	3.5
Proportion of invalid data for malicious vehicles	20%-40%

To simulate real-world conditions, we use the Simulation of Urban Mobility (SUMO) traffic simulator to generate realistic vehicle mobility patterns based on a real-world map of Chengdu. The Intelligent Driver Model (IDM) is used to simulate realistic vehicle behavior, including acceleration, deceleration, and lane changes. We also incorporate variable speed limits to mimic real-world traffic regulations. The dataset used for trust calculation is derived from real-world traffic data collected from the Chengdu traffic management system. The dataset provides authentic and credible behavioral inputs for the vehicles in the simulation experiments, thereby supporting the entire trust model evaluation. Subsequent simulations are conducted based on the trust assessment results generated therefrom, rather than relying on artificially preset theoretical distributions (such as uniform or normal distributions). This approach naturally reflects the actual differences in vehicle trust levels within the IoV environment, thereby more realistically simulating the distribution of trust values that attackers would face in real-world scenarios. Table 4 summarizes the key parameters, dataset characteristics, and attack scenarios used in our simulations.

**Table 4:** Simulation Parameters and Settings.

Parameter	Value
Simulator	OMNET + + 6.0.1
Road Network	SUMO-imported real-world map of Chengdu
Vehicle Mobility Model	Intelligent Driver Model (IDM) with variable speed limits
Simulation Time	3600 s
Trust Update Interval	10 s
Dataset	Real-world traffic data collected from Chengdu traffic management system
Attack Scenario 1: False Recommendations	Malicious vehicles provide incorrect recommended trust values
Attack Scenario 2: Collusion Attack	Malicious vehicles collaboratively provide highly consistent but false recommendation information
Attack Scenario 3: Trust Link Attack	Malicious vehicles attempt to correlate trust values

### Analysis of simulation results

1) Balancing vehicle trust availability with privacy protection.

In this paper, we simulate the relationship between the maximum offset trust $\:Erro{r}_{max}$, the trust discrimination ability $\:\eta\:$ of the vehicle, and the DP privacy budget $\:\epsilon\:$.

**Fig. 6:**
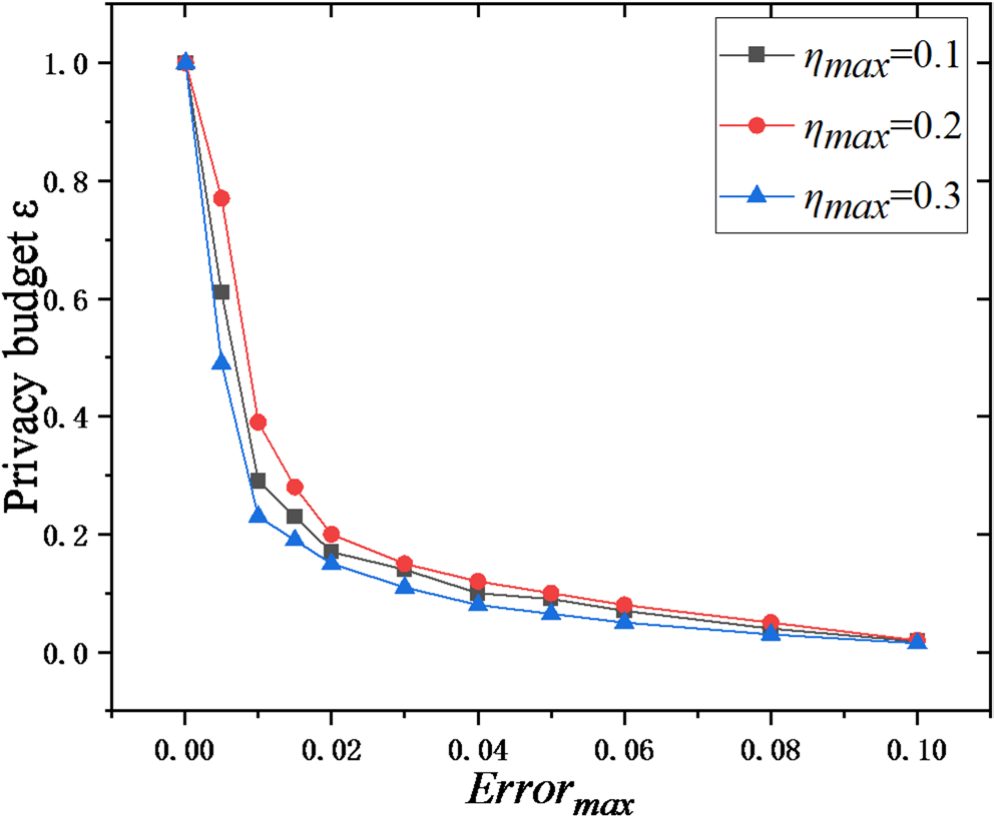
Relationship between *Error*_*max*_, $\:\eta\:$ and $\:\epsilon\:$.

As shown in Fig. 6, there is a negative correlation between $\:\epsilon\:\:$and $\:Erro{r}_{max}$ for a fixed vehicle trust loss probability. A larger $\:Erro{r}_{max}$ means a larger perturbation is generated, resulting in a larger trust bias, a smaller privacy budget$\:\:\epsilon\:$, and a greater degree of privacy protection for the trust value at this time. In addition, we can see from the Fig. 6 that the trust deviation increases rapidly when the $\:\epsilon\:$ decreases below 0.2. It indicates that a critical point is reached at this point. And adding additional perturbations will lead to a significant decrease in the availability of trust values. Meanwhile, as the $\:\eta\:$ increases, the $\:\epsilon\:$ decreases, supporting the addition of more perturbations to the trust set.

2) The identity privacy protection capabilities.

In this paper, we simulate and verify the identity privacy protection capability of the DITDP algorithm by defining two main measures to be tested, namely the identity tracing rate *ITR* and the identity misjudgment rate *IMR*.

*ITR* is used to quantify a scheme’s ability of protect identity privacy, namely, the ability to resist trust link attacks. If the scheme has a high identity tracking rate, its ability to resist link attacks is weak, and the vehicle is at greater risk of identity compromise under this scheme. Conversely, the scheme is more capable of protecting vehicle identity privacy with published trust values. Let $\:nu{m}_{at}$ denote the number of vehicle identities initiated for tracking and $\:nu{m}_{ct}$ denote the number of successfully tracked vehicle identities, then $\:ITR$ can be denoted as $\:ITR=nu{m}_{ct}/nu{m}_{at}$.

In the experiments presented in this paper, the vehicles will change their aliases in a local mixing zone. The attacker traces the target by launching a trust link attack. Specifically, the attacker records a sequence of observations. For each vehicle *V*_*j*_ using the pseudonym *PID*_*j*_(*t*) at time *t*, the attacker observes the corresponding perturbed aggregated trust value AT*_j_(*t*) released by the RSU. When a vehicle changes its pseudonym from *PID*_*j*_(*t*) to *PID*’_*j*_(*t* + Δ*t*) at time *t* + Δ*t*, the attacker attempts to link them. The attacker maintains a candidate set *S*, which contains all observed < pseudonym, trust value> pairs within a recent time window. The linkage process is executed according to the following rules:

(1) The attacker calculates the absolute difference between the newly observed trust value *AT*’_*j*_(*t* + Δ*t*) (under the new pseudonym) and all historically observed trust values *AT*_*k*_(*t*) in the candidate set *S*.

(2) A link is established if and only if there exists a unique​ historical record <*PID*_*k*_(*t*), *AT*_*k*_(*t*)> ∈ *S* that simultaneously satisfies the following two conditions: |*AT*’_*j*_(*t* + Δ*t*) - AT*_k_(*t*)| is the minimum among all differences in the candidate set *S*; and this minimum distance is less than a predefined threshold, e.g., 0.1 * *τ* (where *τ* is the trust threshold).

(3) If multiple historical records satisfy the conditions, or if none do, the attack for this specific pseudonym change event is considered a failure.

*IMR* is used to measure the availability of vehicle trust values after perturbation processing. If the vehicles in the scheme have a low *IMR*, their published vehicle trust values are more trustworthy. On the contrary, the availability of vehicle trust values is low and there are more cases of misjudging a trusted vehicle as a malicious vehicle. The $\:IMR$ can be denoted as $\:IMR=nu{m}_{a}/nu{m}_{m}$. Where $\:nu{m}_{a}$ is the number of making trustworthy judgments. And the number of those judged to be inconsistent with the true identity of the vehicle based on the checked trust value be $\:nu{m}_{m}$.

**Fig. 7:**
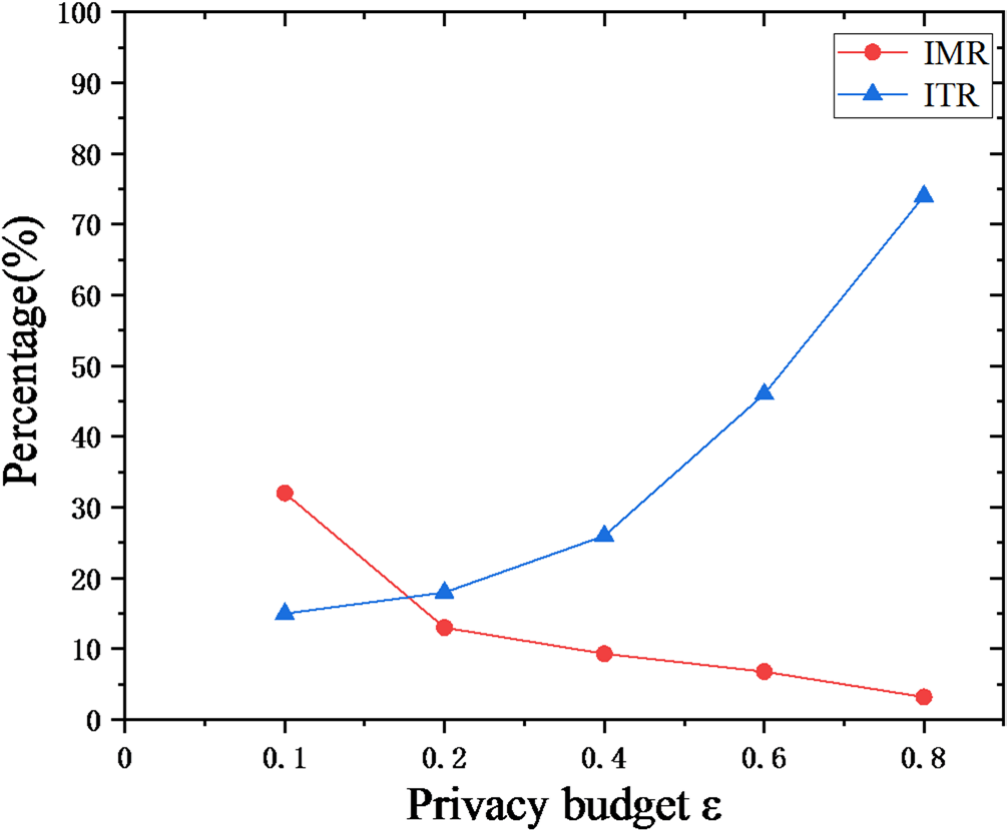
Relationship between privacy pudget $\:\epsilon\:$ and *IMR*, ITR.

Next, the impact of the DITDP algorithm on ITR and *IMR* with different privacy budgets is verified, as shown in Fig. 7. Here, the privacy budget $\:\epsilon\:$ is set to 0.1, 0.2, 0.4, 0.6, and 0.8. The ITR increases as the privacy budget $\:\epsilon\:$ increases. And the ITR curve becomes steeper and steeper, reaching 74.1% when $\:\epsilon\:$ is taken to be 0.8. The larger the privacy budget $\:\epsilon\:$, the smaller *IMR*. It means that the closer the published trust value is to the true value, the easier it is to be tracked by attackers. The *IMR* decreases as the privacy budget increases and the decrease tends to be flat, reaching a high of 34.9% when$\:\:\epsilon\:$ is taken to be 0.1. Moreover, it is shown that when the privacy budget $\:\epsilon\:$ is small, especially less than 0.2, the added perturbation has a larger impact on the availability of trust values and on the vehicle identity judgment. Therefore, the different effects of the privacy budget $\:\epsilon\:$ on ITR and IMR demonstrate that privacy-preserving treatment of trust can lead to conflicting properties with the availability of trust values.

Overall, a smaller value of *ε* provides stronger privacy guarantees but also introduces greater perturbation to the trust values, potentially increasing the *IMR*. Conversely, a larger value of *ε* provides weaker privacy guarantees but reduces the distortion of trust values, leading to a lower *IMR*. Therefore, a careful selection of *ε* is required to balance the trade-off between privacy protection and trust accuracy.

3) Identity privacy protection capability comparison.

**Fig. 8:**
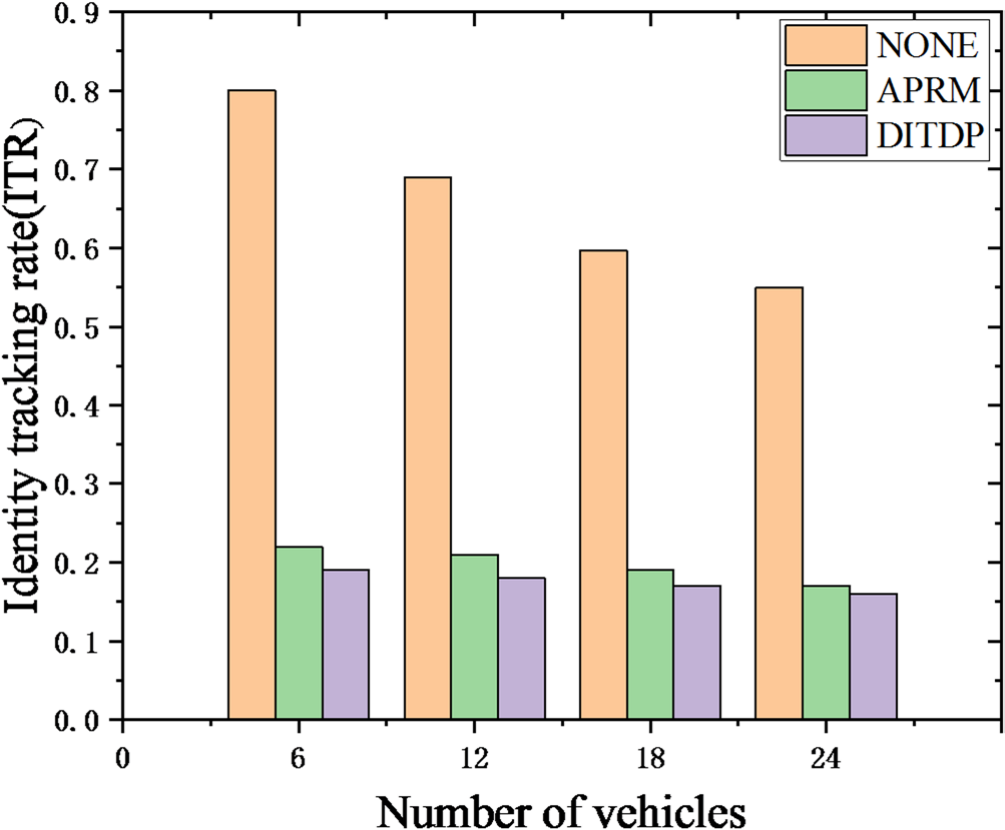
Identity tracking rate (ITR).

As far as we know, the trust link attack is proposed for the first time in this paper, and no other articles have considered this issue. Therefore, to better prove the identity privacy protection ability, this experiment compares DITDP algorithm with the scheme without trust value privacy treatment (NONE), and the APRM algorithm^26^. Our experiment sets different numbers of vehicles as 6, 12, 18, and 24 for the local area, as shown in Fig. 8. The NONE scheme has the highest *ITR* value. Because it does not privacy-protect the published trust values, which are the true trust values of the vehicles, leading the attacker to easily perform vehicle tracking by querying the trust values multiple times. Of course, it can also be found from that the *ITR* of each scheme decreases as the number of vehicles increases. The more local vehicles represent the larger the range of trust values of the vehicles. This results in more overlapping the trust values of the vehicles after the DITDP perturbation, making it more difficult for the attacker to distinguish the identity of the vehicles. In all, the overall identity tracking rate ITR of the DITDP algorithm (with an average reduction of 10.5% in *ITR*) is lower than that of the APRM algorithm. Thus, it implies that the DITDP algorithm has higher privacy protection for vehicle identities.

**Fig. 9:**
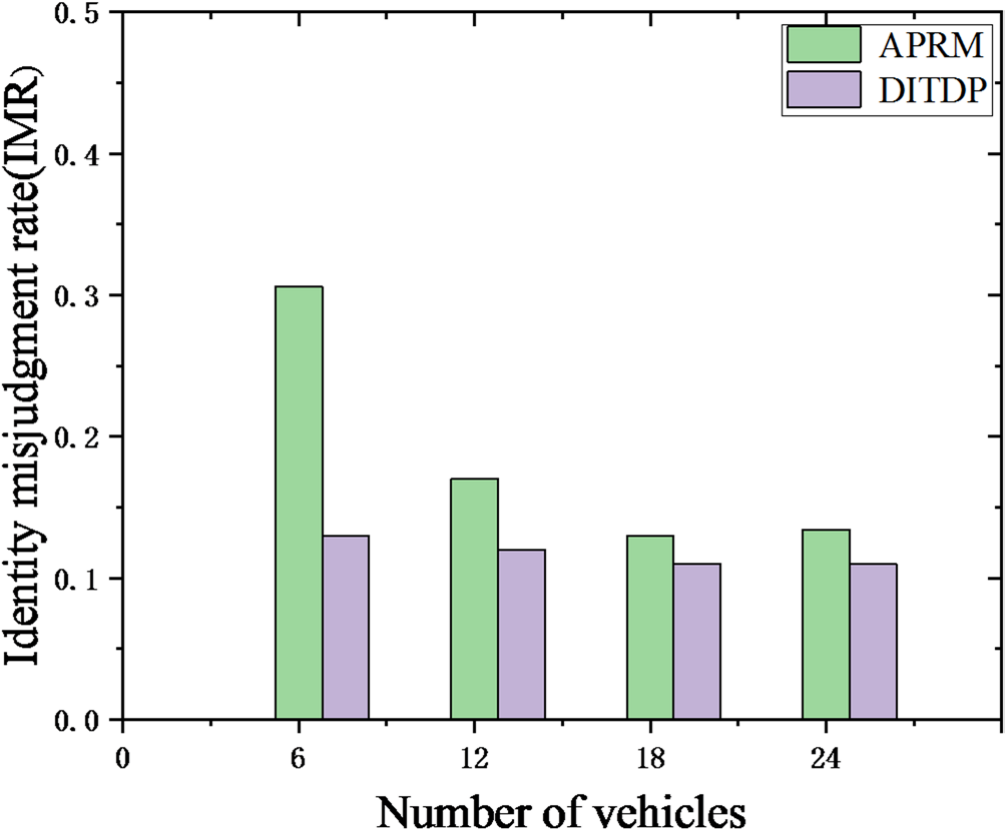
Identity misclassification rate (IMR).

Since NONE does not apply privacy protection to the trust values and publishes the true vehicle trust, there is no such metric as the identity misclassification rate. Therefore, we compare the IMR performance between DITDP and APRM, as shown in Fig. 9. When the number of vehicles is small, the IMR is large, which leads to many wrong judgments and affects the normal communication of vehicles. The IMR of the DITDP algorithm remains stable with the number of vehicles, and the number of vehicles has little effect on the IMR. This is because the identity privacy protection capability provided by the DITDP algorithm is independent of the surrounding vehicles. It is determined by the perturbation mechanism. We can get that the overall IMR of the DITDP algorithm is lower than that of the APRM algorithm, with an overall average reduction of 18.6%. It indicates that the trust value after differential privacy processing of the DITDP algorithm has less influence on whether the vehicle is judged to be trustworthy. And the trust value is more usable and can be used more safely by vehicles for trust decisions in DITDP.

Additionally, to quantitatively assess the reliability of performance differences between the DITDP algorithm and comparison schemes, rather than merely observing average trends, we conducted statistical significance tests on the experimental results of ITR and IMR.

Specifically, we use independent sample *t-*test to analyze the differences in ITR and IMR metrics between DITDP and the APRM scheme. The null hypothesis for this test is set as: there is no significant difference in the mean values of the metrics between DITDP and APRM. We chose a 95% confidence level (α = 0.05) as the criterion for determining statistical significance. The results of the significance tests are shown in the Table 5. The *t*-value represents the size of the mean difference relative to the within-group variation, while the *p*-value indicates the probability of observing the current or greater difference when the null hypothesis is true.

**Table 5:** Independent Sample t-Test Results.

Performance Metrics	t	*p*	95% Confidence Interval
ITR​	-5.82	< 0.001​	[-14.1%, -6.9%]
IMR	-8.45	< 0.001	[-23.1%, -14.1%]

For the ITR Metric, the *p*-value obtained from the test is far less than 0.001, leading to a strong rejection of the null hypothesis. The 95% confidence interval is [− 14.1%,−6.9%], with the entire interval lying in the negative range and not containing zero. This indicates that the reduction in ITR brought about by the DITDP algorithm (i.e., the enhancement of privacy protection) is highly statistically significant. It means 95% confident that the true effect lies between a reduction of 6.9% and 14.1%. For the IMR Metric, similarly, the *p*-value is less than 0.001, with a confidence interval of [− 23.1%,−14.1%], which also does not contain zero. This demonstrates that DITDP significantly reduces the identity misclassification rate (i.e., enhances trust availability) with high statistical significance, and the estimated true improvement lies between 14.1% and 23.1%.

In a word, the combined IMR and ITR comparison results show that the DITDP algorithm has higher identity privacy protection with guaranteed trust availability.

## Conclusion

To address the conflict between vehicle trust and identity privacy in the connected domain, we propose a differential privacy-based algorithm to manage vehicle identity trust in the connected vehicle domain. First, an identity trust assessment model is proposed and a multivariate mathematical model is designed to complete the quantification of direct trust, synthesis of recommended trust, and computation of aggregated trust for vehicles. Vehicle trust values are multiplexed via distributed quantification of trust for vehicles and centralized management of aggregated trust for entities. Then, based on this trust assessment model, we propose identity trust management algorithms including dynamic trust evaluation and identity privacy protection. Dynamic trust assessment corrects the conflicting nature between the vehicle trust components and dynamically updates the vehicle trust values in the time domain. Identity privacy protection provides privacy protection for vehicle identity through differential privacy to achieve trust non-distinguishability and resist trust link attacks to track vehicle identity through vehicle trust while ensuring the availability of vehicle trust value data. Finally, we demonstrate through simulations that the proposed algorithm performs well in terms of identity protection, trust availability, etc., with an average reduction of 10.5% in identity tracing rate and 18.6% in identity misclassification rate.

Although, in an ideal scenario, DITDP can find a perfect balance between trust and privacy, maximizing the utility of the algorithm and identifying the balance point can be challenging in different real-world situations. Excessive trust perturbation can lead to inaccurate trust evaluations, potentially impacting the effectiveness of the system in identifying malicious vehicles. Furthermore, the computational complexity of the trust component correction and Staircase perturbation mechanisms can be a limitation for resource-constrained RSUs. While our experiments primarily focused on evaluating the privacy-preserving capabilities of DITDP, it is also important to consider its performance as a standalone trust prediction model, without the differential privacy component. In such a scenario, the computational overhead would be reduced, and the trust accuracy would likely improve. However, the vulnerability to trust link attacks would also increase. A full evaluation of this trade-off is a subject for future research.

Future research will focus on several key areas:

Optimizing the Privacy-Utility Trade-off: Developing adaptive mechanisms for dynamically adjusting the privacy budget (*ε*) based on network conditions and message types to minimize the impact on trust accuracy.

Integrating Machine Learning for Trust Prediction: Incorporating machine learning techniques to improve the accuracy of trust prediction and reduce the reliance on historical data, making the system more robust against dynamic changes in vehicle behavior.

Real-World Testing: Conducting extensive real-world testing in vehicular environments to evaluate the performance of DITDP under realistic conditions and identify potential deployment challenges.

## Data Availability

Data openly available in a public repository. The data that support the findings of this study are openly available in file “VANET-Intra domain” at https://drive.google.com/file/d/1LkI2bNL5-UImxD7Hq_3tNmCVb4tUJZgJ/view? usp=sharing.

## References

[1] Zhu, C. C. et al. Time-Driven and Privacy-Preserving Navigation Model for Vehicle-to-Vehicle Communication Systems. *IEEE Trans. Veh. Technol.* **72** (7), 8459–8470 (2023).

[2] Hbaieb, A., Ayed, S. & Chaari, L. A survey of trust management in the Internet of Vehicles. *Comput. Netw.* **203**, 108558 (2022).

[3] Bagga, P. et al. On the Design of Mutual Authentication and Key Agreement Protocol in Internet of Vehicles-Enabled Intelligent Transportation System. *IEEE Trans. Veh. Technol.* **70** (2), 1736–1751 (2021).

[4] Wang, C. et al. BPS-V: A Blockchain-Based Trust Model for the Internet of Vehicles with Privacy-Preserving. *Ad Hoc Netw.* **163**, 103566–103578 (2024).

[5] Razafimanjato, M., Saad, M. M. & Kim, D. Blockchain-based trust management systems in the Internet of Vehicles: A comprehensive survey. *ICT Express*. **7** (3), 30621–20642 (2025).

[6] Han, H. et al. Trust management scheme of IoV based on dynamic sharding blockchain. *Electronics* **13** (6), 1016–1033 (2024).

[7] Ren, Y. A dynamic trust evaluation scheme based on cross-domain trust inheritance for VANETs. *J. Netw. Comput. Appl.* **195**, 103236–103250 (2025).

[8] Sehar, N. et al. Blockchain enabled data security in vehicular networks. *Sci. Rep.* **13** (1), 4412–4427 (2023). 36932131 10.1038/s41598-023-31442-w PMC10023690

[9] Cui, J. et al. RSMA: Reputation System-Based Lightweight Message Authentication Framework and Protocol for 5G-Enabled Vehicular Networks. *IEEE Internet Things J.* **6** (4), 6417–6428 (2019).

[10] Yang, X. & Li, C. Vehicle Behavior Prediction Based Authentication Scheme in IoV. *Comput. Eng.* **47** (1), 129–138 (2021).

[11] Wang, J. et al. RPRep: A robust and privacy-preserving reputation management scheme for pseudonym-enabled VANETs. *Int. J. Distrib. Sens. Netw.* **12** (3), 234–251 (2016).

[12] Inedjaren, Y. et al. Blockchain-based distributed management system for trust in VANET. *Veh. Commun.* **30**, 100350 (2021).

[13] Wang, S., Hu, Y. & Qi, G. Blockchain and deep learning based trust management for Internet of Vehicles. *Simul. Model. Pract. Theory*. **120**, 102627 (2022).

[14] Liu, J. et al. CPAHP: Conditional Privacy-Preserving Authentication Scheme With Hierarchical Pseudonym for 5G-Enabled IoV. *IEEE Trans. Veh. Technol.* **72** (7), 8929–8940 (2023).

[15] Maurya, C. & Chaurasiya, V. K. Efficient Anonymous Batch Authentication Scheme With Conditional Privacy in the Internet of Vehicles (IoV) Applications. *IEEE Trans. Intell. Transp. Syst.* **24** (9), 9670–9683 (2023).

[16] Cheng, G. et al. Conditional Privacy-Preserving Multi-Domain Authentication and Pseudonym Management for 6G-Enabled IoV. IEEE Trans. Inf. Forensics Secur. 19, 10206–10220 (2024).

[17] Mahmoud, H. et al. A Framework for Decentralized, Real-Time Reputation Aggregation in IoV. *IEEE Internet Things Magazine*. **6** (2), 44–48 (2023).

[18] Parameswarath, R. P., Gope, P. & Sikdar, B. A Privacy-Preserving Authenticated Key Exchange Protocol for V2G Communications Using SSI. *IEEE Trans. Veh. Technol.* **72** (11), 14771–14786 (2023).

[19] Hou, W. et al. Lightweight and Privacy-Preserving Charging Reservation Authentication Protocol for 5G-V2G. *IEEE Trans. Veh. Technol.* **72** (6), 7871–7883 (2023).

[20] Miao, Q. et al. A 3 C Authentication: A Cross-Domain, Certificateless, and Consortium-Blockchain-Based Authentication Method for Vehicle-to-Grid Networks in a Smart Grid. *Symmetry* **16** (3), 336 (2024).

[21] Li, C. et al. CIM: CP-ABE-based identity management framework for collaborative edge storage. *Peer-to-Peer Netw. Appl.* **17** (2), 639–655 (2024).

[22] Ruan, W. et al. A Double-Layer Blockchain Based Trust Management Model for Secure Internet of Vehicles. *Sensors* **23** (10), 4699 (2023). 37430611 10.3390/s23104699 PMC10223821

[23] Dempster, A. P. Upper and Lower Probabilities Induced by a Multivalued Mapping. *Annals Math. Statistic*. **38** (2), 325–339 (1967).

[24] Jin, F., Guan, Y., Liu, J. & Zhou, L. Bayesian inference and minimum consensus adjustment process for multi-attribute large-scale group decision-making in social networks. *Inform. Fusion*. **126**, 103537–103558 (2026).

[25] Andrés, M. E. et al. Geo-indistinguishability: Differential privacy for location-based systems. *Proceedings of the 2013 ACM SIGSAC conference on Computer & communications security*, 901–914. ( New York, USA, 2013).

[26] Wang, J. et al. RPRep: A robust and privacy-preserving reputation management scheme for pseudonym-enabled VANETs. *Int. J. Distrib. Sens. Netw.* **12** (3), 234–251 (2016).

